# Breeding oat for resistance to the crown rust pathogen *Puccinia coronata* f. sp. *avenae*: achievements and prospects

**DOI:** 10.1007/s00122-022-04121-z

**Published:** 2022-06-04

**Authors:** R. F. Park, W. H. P. Boshoff, A. L. Cabral, J. Chong, J. A. Martinelli, M. S. McMullen, J. W. Mitchell Fetch, E. Paczos-Grzęda, E. Prats, J. Roake, S. Sowa, L. Ziems, D. Singh

**Affiliations:** 1 School of Life and Environmental Sciences, Faculty of Science, The University of Sydney, Sydney, Australia; 2 Department of Plant Sciences, University of the Free State, P.O. Box 339, Bloemfontein, 9300 South Africa; 3 Swift Current Research and Development Centre, Agriculture and Agri-Food Canada, Swift Current, Canada; 4 Morden Research and Development Centre, Agriculture and Agri-Food Canada, Morden, Canada; 5 Department of Crop Science, Agronomy School, Federal University of Rio Grande Do Sul (UFRGS), Av. Bento Gonçalves, 7712, Porto Alegre, RS 91501-970 Brazil; 6 Department of Plant Sciences, North Dakota State University, Fargo, ND 58105-5051 USA; 7 Brandon Research and Development Centre, Agriculture and Agri-Food Canada, Brandon, Canada; 8 Institute of Plant Genetics, Breeding and Biotechnology, University of Life Sciences in Lublin, 20-950 Lublin, Poland; 9 CSIC-Institute for Sustainable Agriculture, Avda. Menéndez Pidal s/n. , 14004 Córdoba, Spain

## Abstract

Crown rust, caused by *Puccinia coronata* f. sp. *avenae* (*Pca*), is a significant impediment to global oat production. Some 98 alleles at 92 loci conferring resistance to *Pca* in *Avena* have been designated; however, allelic relationships and chromosomal locations of many of these are unknown. Long-term monitoring of *Pca* in Australia, North America and elsewhere has shown that it is highly variable even in the absence of sexual recombination, likely due to large pathogen populations that cycle between wild oat communities and oat crops. Efforts to develop cultivars with genetic resistance to *Pca* began in the 1950s. Based almost solely on all all-stage resistance, this has had temporary benefits but very limited success. The inability to eradicate wild oats, and their common occurrence in many oat growing regions, means that future strategies to control *Pca* must be based on the assumption of a large and variable prevailing pathogen population with high evolutionary potential, even if cultivars with durable resistance are deployed and grown widely. The presence of minor gene, additive APR to *Pca* in hexaploid oat germplasm opens the possibility of pyramiding several such genes to give high levels of resistance. The recent availability of reference genomes for diploid and hexaploid oat will undoubtedly accelerate efforts to discover, characterise and develop high throughput diagnostic markers to introgress and pyramid resistance to *Pca* in high yielding adapted oat germplasm.

**Supplementary Information:**

The online version contains supplementary material available at 10.1007/s00122-022-04121-z.

## Introduction

“*The extremely variable heteroecious fungus,* Puccinia coronata*, attacks many Gramineae and causes economically important crown rust of oat. Such a high level of specialization is exhibited on the 190 species of susceptible Gramineae and 59 species of* Rhamnus *and 6 other genera of* Rhamnaceae *and* Eleagnaceae *that a satisfactory sub-specific classification of the pathogen has still to be devised. Despite this difficulty, breeding programs have developed temporarily useful commercial resistant varieties for the major oat-growing areas of the world. The use of multiline resistant cultivars seems particularly promising for control. This disease thus illustrates the continuing race between the plant breeder and the pathogen*”. This introductory paragraph from a monograph on crown rust of oats and grasses by Simons (1970) provides deep insight into the status of knowledge and progress in breeding oat for resistance to the destructive crown rust disease in 1970.

Among cereals, oat ranks seventh in the world in terms of production, after wheat, maize, rice, barley, sorghum and millet. This ranking is achieved largely through the use of improved cultivars of *Avena sativa* for livestock feed and human consumption, and to some extent cultivars of *A. strigosa* grown mostly in South America (Suttie and Renolds 2004). Awareness of the nutritional and health benefits of oat continues to increase, especially since the discovery of phytoalexins such as avenanthramides (Collins 1989) in oat groats. These phenolic compounds are reported to possess antioxidant (Emmons and Peterson 1999) and cholesterol (LDL) lowering properties (Chen et al. 2004). Minimising losses to diseases will be critical in meeting projected increases in future global demand for cereals such as oat. Crown rust (caused by *Puccinia coronata* f. sp. *avenae*; *Pca*) and stem rust (caused by *Puccinia graminis* f. sp. *avenae*; *Pga*) occur in all major oat growing regions of the world and have caused significant yield losses in oat crops. *Pca* is widespread globally (Savile 1984) and is considered one of the most important oat diseases and an important factor limiting oat production world-wide (Harder and Haber 1992; Sebesta et al. 2003; Nazareno et al. 2018). It was considered by Leonard et al. (2004) as the most serious threat to oat production in North America, with records of epidemics dating back more than 100 years. From 1918 to 1930, annual estimated losses in the USA averaged greater than 13.7 million bushels (Murphy 1935). Simons and Murphy (1961) reported annual losses of up to 30% in years when crown rust was severe. Severe damage due to *Pca* has also occurred in coastal areas of Australia in years favourable to the disease (Waterhouse 1952; Luig 1985). Grain yields are negatively correlated with crown rust severity, and may be reduced by as much as 50% in susceptible cultivars (Leonard and Martinelli 2005). In addition to causing reductions in grain yield, severe crown rust can also reduce the yield and quality of forage (Simons 1985).

A great deal of effort has been made to investigate the genetics of resistance to *Pca* in *Avena*, quite possibly more than any other pathogen of oat, with a view to developing crown rust resistant oat cultivars. However, the lack of a physical map for the oat genome for many years limited advances in the genetics of resistance to *Pca*. Some 98 alleles at 92 loci that confer resistance to *Pca* in *Avena* have been catalogued and accepted, however the chromosomal location of many of these genes remains unknown. In addition, studies on the pathogenicity of *Pca* on these alleles have shown that virulence for most exists, making breeding for resistance to this rust pathogen very challenging. Tellingly, as quoted above, Simons (1970) stated that “*breeding programs have developed temporarily useful commercial resistant varieties*”, a testament to the propensity of *Pca* to change and evade detection by resistance genes. This review summarises the current state of knowledge of the genetic basis of resistance to *Pca* in oat, how it has been applied to resistance breeding, and looks to future advances that we believe will lead to more sustained and environmentally sound approaches to control this globally damaging pathogen.

## Advances in oat genomics

*Avena sativa* has a large genome (ca. 12.5 Gb) comprising 21 chromosome pairs from three separate ancestral genomes, with the genome constitution of AACCDD. Physical rearrangements among the ancestral genomes (e.g. Chen and Armstrong 1994; Leggett and Markhand 1995) have impeded the development of linkage maps for *A. sativa*, and at times have made the assignment of loci to chromosomes very difficult. The first oat consensus map was developed by Oliver et al. (2013), based on 985 SNPs assayed on 390 recombinant inbred lines (RILs) derived from six hexaploid oat biparental populations and SNP deletion analysis in a set of monosomic stocks. This map provided substantial improvements over previous maps because of low error rates in the scoring of SNP markers, and the joint mapping of markers assayed in parallel across multiple populations. Further improvements were made by integrating high-density SNPs discovered via genotype-by-sequencing (GBS) (Tinker et al. 2014).

In a further attempt to develop a single high density consensus linkage map representative of the majority of commonly grown oat cultivars, Chaffin et al. (2016) not only saturated the map with additional populations and genetic markers but also corrected errors in the previous map and gained a better understanding of biological deviations among existing maps. Homoeologous regions among oat chromosomes and matches to orthologous regions of rice (*Oryza sativa*) revealed that the hexaploid oat genome is highly rearranged compared to the diploid genome as a consequence of frequent translocation among chromosomes and such subgenome rearrangements probably accounted for the failure of certain linkage groups to correspond to the consensus map. In other advances in oats genomics, Yan et al. (2016) performed high-density marker profiling and genome analysis of 27 oat species and further identified ancestral origins of 21 mapped chromosomes in hexaploid oat, and deduced the likely pathway from which hexaploid oat originated by sequential polyploidization events. In a further de novo GBS analysis of 4,657 accessions of cultivated oat, Bekele et al. (2018) discovered 164,741 tag-level genetic variants comprising 241,224 SNPs that were used to expand marker density of the existing oat consensus map by the addition of more than 70,000 GBS loci (Tinker et al. 2016). This high-density marker system was used to construct the first oat haplotype map.

GBS marker analysis by Latta et al. (2019) allowed additional improvement in the maps of diploid A genome (*A. strigosa* x *A. wiestii*, 2n = 14), and tetraploid AB genome (*A. barbata* 2n = 28). Seven linkage groups in the tetraploid showed considerably greater homology and synteny with the A genome diploids than did the other seven, suggesting an allopolyploid hybrid origin of *A. barbata* rather than autopolyploid genome doubling within a single species.

Bekele et al. (2020) enhanced GBS in oat by developing the targeted assay ‘Rapture’, based on an additional bait-based capture of specific DNA fragments representing close to 10 K loci. The assay accomplished deeper sequence coverage of target markers by including only those fragments that provided effective polymorphic markers. The authors argued that Rapture was cost-effective (40% less than GBS) and consistently outperformed the GBS or SNP assays when filtering markers at 80% completeness or greater, even though the total number of reads per sample of Rapture was only a quarter of that of GBS.

Although accomplishment of a full genome assembly of hexaploid oats is extremely challenging because of its highly repetitive large genome size with numerous major intra – and intergenomic arrangements, Maughan et al. (2019) developed fully annotated, chromosome scale assemblies for the extant progenitor species of the As- and Cp-subgenomes, *A. atlantica* and *A. eriantha*, respectively. These genome assemblies span 3.69 Gb with an N50 of 513 Mb (*A. atlantica*) and 3.78 Gb with an N50 of 535 Mb (*A. eriantha*) annotating approximately 50 K gene models in each species that include ~ 3 K resistance gene analogs. By systematically comparing consensus maps and analysing syntenic relationships with other cereals and homeology within oat, the authors found orthologous relationships within *Pooideae* and subgenome origins for each of the 21 hexaploid linkage groups.

In 2020, Pepsico in partnership with several public and private research organizations unveiled the first ever *A. sativa* genome assembly v1 for use in open-source applications (https://wheat.pw.usda. gov/GG3/graingenes_downloads/oat-ot3098-pepsico). Based on the oat genotype OT3098, this assembly represents a major breakthrough in oat genomics research and will open new horizons in spurring genetic improvement of traits of interest in oat including high throughput mapping and cloning of crown rust resistance genes. Jiang et al. (2021) predicted the chromosomal distribution of tandem repeats of all the 21 individual chromosomes by analysing data from the OT3098 reference assembly v1. Eight new oligonucleotide probes were then designed for non-denaturing fluorescence in situ hybridization, which were used along with 11 existing probes for chromosome karyotyping on mitotic metaphase spreads of six different species (*A. brevis*, *A. nuda*, *A. wiestii*, *A. ventricosa*, *A. fatua* and *A. sativa*). Breakage points in some chromosome translocations in *A. sativa* were also identified.

An advanced version (v2) of the OT3098 assembly has also been released, incorporating gap filling as well as reorientation/flipping of certain chromosomes. As the entire chromosome will be inverted/flipped, the positions for a subset of the chromosomes will change but this chromosome orientation will ensure consistent nomenclature of current and future *A. sativa* assemblies.

A new convention for numbering chromosomes in oat was accepted by the International Oat Nomenclature Committee (https://protect-au.mimecast.com/s/XLulC2xMQzip25rzMcnoWMY?domain=wheat.pw.usda.gov). The convention is based on an in-depth phylogeny-based analysis that established the presence of conserved core regions with collinearity across *Avena*, barley and wheat, using a phylogeny-based numbering and orientation system in which hexaploid, tetraploid, and diploid *Avena* chromosomes are numbered and oriented based on an ancestral core region that is consistent with the Triticeae. In the remainder of this manuscript, the IONC accepted chromosome nomenclature is provided for any locus with a published genetic location using previous systems, using the format [**chr. xx]**.

### The genetics and durability of resistance to rust in cereals

Genetic analysis of disease resistance in plants began when Biffen (1905) demonstrated Mendelian inheritance of stripe rust resistance in wheat. Since then, many genes conferring resistance in plants have been characterised, and the inheritance of pathogenicity (virulence/ avirulence) has been investigated in many plant pathogens including several that cause rust in cereals. Based on studies of the genetic basis of the interaction between flax and the flax rust pathogen, Flor (1955) developed the gene-for-gene hypothesis, which has provided an important framework for much of the genetic work on plant: pathogen interactions since.

Resistance to many plant pathogens has been defined on the basis of genetics or function. In genetic terms, it is generally defined on the basis of inheritance: oligogenic, being controlled by one or few genes of major effect; polygenic, being controlled by many genes of individually small phenotypic effect. In functional terms, resistance is defined based on epidemiological (e.g. slow rusting, rate reducing) or developmental (e.g. all-stage resistance (ASR), also referred to as seeding or major gene resistance versus adult plant resistance (APR), also often referred to as minor gene resistance) criteria.

Most of the rust resistance genes in cereals that have been catalogued confer ASR. The process of identifying and designating rust resistance loci in cereals involves initially showing that a given resistance is distinct from previously catalogued resistances based on rust isolate specificity and/or genomic location, and then showing that the new resistance is either a locus independent of or allelic to previously catalogued alleles prior to a gene designation being assigned (see McIntosh et al. 1995). A critical part of this process is the nomination of a reference genetic stock carrying the gene, which can be accessed by the international scientific community for further study and use in resistance breeding. In studies where more than one resistance gene is found in a specific genotype, single gene progeny should be generated for each and identified as single gene reference stocks.

The concept of durable resistance was introduced in 1978 by Dr Roy Johnson, following the failure of resistance to stripe rust in some English winter wheats and the continued effectiveness of resistance in others (Johnson 1978). Genetic studies since then have dissected durable resistance to rust in several wheat genotypes, and genes conferring either ASR or APR to rust are being isolated from wheat and barley genomes. These studies have shown a greater tendency for lack of durability in ASR genes encoding nucleotide-binding and leucine-rich repeat (NLR) receptor proteins compared to other non-NLR-encoding resistance genes. Of the 17 ASR NLR-encoding rust resistance genes isolated from wheat (Zhang et al. 2020) and two isolated from barley (Dracatos et al. 2019; Chen et al. 2021) to date, only the ASR stem rust resistance gene *Sr26* (Zhang et al. 2021) has been deployed widely and remained effective, being used in Australian agriculture in more than 30 wheat cultivars since it was first deployed in the cultivar Eagle in 1969 (Park 2007).

Two loci, *Lr34*/*Yr18*/*Sr57* (Krattinger et al. 2009), and *Lr67*/*Yr46*/*Sr55* (Moore et al. 2015), confer APR and encode an ATP-binding cassette transporter, and a protein that has lost hexose transport function, respectively. The loci *Lr34*/*Yr18*/*Sr57* and *Lr67*/*Yr46*/*Sr55*, along with *Lr46*/*Yr29*/*Sr58*, confer resistance against all three rust pathogens, and are associated with a linked morphological trait known as leaf tip necrosis. Experience to date suggests that these pleiotropic APR genes have some intrinsic durability, and that combinations of multiple effective resistance genes contribute to durability by lowering the chance of virulence matching gene combinations developing. Pleiotropic APR genes have been used to great effect in conjunction with ASR genes, providing backbone resistance that may also contribute to increasing the durability of ASR genes (Park 2015).

### Resistance to *Puccinia coronata* f. sp. *avenae* in *Avena*

When catalogued, genes conferring resistance to *Pca* in oat are given the designation “*Pc*”. Although Simons et al. (1978) used the designation “*Pc-*”, most publications since have used simply “*Pc*”. At this time, 92 loci that confer resistance to *Pca* in *Avena* have been designated and accepted (*Pc1*-*Pc85*, *Pc91*-*Pc96*, *Pc98*). Six loci are reported as conferring APR (*Pc27*, *Pc28*, *Pc69*, *Pc72*, *Pc73*, *Pc74*) and the remaining 86 conferring ASR. Multiple alleles at five loci have been reported: *Pc2* (*Pc2*, *Pc2b*), *Pc3* (*Pc3*, *Pc3c*), *Pc4* (*Pc4*, *Pc4c*), *Pc6* (*Pc6*, *Pc6c*, *Pc6d*) and *Pc9* (*Pc9* and *Pc9c*). Four pairs of complementary genes have been designated (*Pc3* + *Pc4*, *Pc3c* + *Pc4c*, *Pc7* + *Pc8*, *Pc24* + *Pc25*; Tables 1 and 2). The *Pc* genes designated to date have been found in cultivated and wild hexaploid oat, *A. sativa* and *A. sterilis,* respectively, and other related AACCDD sub-species, *A. strigosa* (AA, diploid)*, A. abyssinica* (AABB, tetraploid)*,* and *A. magna* (CCDD, tetraploid). *A. sterilis* has proven a particularly rich source of resistance, contributing some 44 of the resistance loci catalogued to date. This may be in part because it is a sexually compatible hexaploid, making it far easier to transfer this resistance into cultivated oat.

**Table 1:** Designated (catalogued) genes conferring resistance to *Puccinia coronata* f. sp. *avenae* in *Avena* for which nominated single gene stocks do not exist

Genes	Species	Original source	Tester line	ASR or APR?
*Pc2, Pc11, Pc12*	*Avena byzantina*	Victoria	Victoria	ASR
Pc2b, Pc6d*	*Avena sativa*	Anthony/Bond//Boone	Anthony/Bond//Boone	ASR
*Pc3c*+*Pc4c, Pc6c, Pc9*	*Avena sativa*	Ukraine	Ukraine	ASR
*Pc4, Pc5*	*Avena byzantina*	Landhafer	Landhafer	ASR
*Pc6, Pc7*+*Pc8, Pc21*	*Avena byzantina*	Santa Fe	Sante Fe	ASR
*Pc15, Pc16, Pc17*	*Avena strigosa*	*A.strigosa* Saia	Saia	ASR
*Pc18, Pc29*	*Avena strigosa*	*A.strigosa* Glabrota	Glabrota	ASR
*Pc19, Pc30, Pc81* through *Pc85*	*Avena strigosa*	*A. strigosa* CI 3815	CI 3815	ASR
*Pc24*+*Pc25, Pc26, ****Pc27****, ****Pc28***	*Avena sativa*	Garry	Garry	ASR/**APR**
*Pc32, Pc33*	*Avena strigosa*	Ceirch Llwyd	Ceirch Llwyd	ASR
Pc40*, Pc41, Pc42, Pc43	*Avena sterilis*	*Avena sterilis* F-83	Avena sterilis F-83	ASR
*Pc60, Pc61*	*Avena sterilis*	*Avena sterilis* PI 287,211	Coker 227, Coker 234	ASR
Pc64*, Pc65, Pc66	*Avena sterilis*	*Avena sterilis* CAV 4248	Makuru//Sun II Pc64	ASR

*Single gene stocks exist for this specific gene (see Table 2)

**a “+” sign between two loci indicates complementarity

**Table 2:** Designated (catalogued) genes conferring resistance to *Puccinia coronata* f. sp. *avenae* in *Avena* species for which a presumed single gene stock has been nominated

Gene	Linkage	Original source	Tester line	ASR or APR?
*Pc1*		*A. byzantina*	Red Rustproof	ASR
*Pc3*+Pc4*		*A. byzantina*	Bond	ASR
*Pc6d*		A*. byzantina*	Trispernia	ASR
*Pc9c*		*A. sativa*	Santa Fe deriv	ASR
*Pc10*		*A. byzantina*	Klein 69B	ASR
*Pc13*		*A. sativa*	Clinton	ASR
*Pc14*		*A. byzantina*	Ascencao	ASR
*Pc20*		*A. abyssinica* CI 7233	CI 7233	ASR
*Pc22*		*A. sativa*	Ceirch du Bach	ASR
*Pc2*		*A. strigosa* CD 3820	CD 3820	ASR
*Pc3*		*A. strigosa* CI 4746	CI 4746	ASR
*Pc34*		*A. sterilis* D-60	D-60	ASR
*Pc35*	*Pc54*	*A. sterilis* D-137	Pendek/ Pc35	ASR
*Pc36*		*A. sterilis* CI 8081	IA D515 or H382	ASR
*Pc3*		*A. strigosa* CD 7994	CD 7994	ASR
*Pc38*	*Pc62, Pc63*	*A. sterilis* CW491-4	Pendek/Pc38	ASR
*Pc39*	*Pc55*	*A. sterilis* F-366	Pendek/Pc39	ASR
*Pc40*		*A. sterilis* F-83	Pendek/Pc40	ASR
*Pc44*	Pc46, Pc50, Pc68, Pc95, PcX**	*A. sativa*	Kyto	ASR
*Pc45*		*A. sterilis* F-169	Pendek/Pc45	ASR
*Pc46*	Pc44, Pc50, Pc68, Pc95, PcX**	*A. sterilis* F-290	Pendek/Pc46	ASR
*Pc47*		*A. sterilis* CI 8081A	Pendek/Pc47	ASR
*Pc48*		*A. sterilis* F-158	Pendek/Pc48	ASR
*Pc49*		*A. sterilis* F-158	A. sterilis F-158	ASR
*Pc50*	Pc44, Pc46, Pc68, Pc95, PcX**	*A. sterilis* CW-486	Pendek/Pc50	ASR
*Pc51*		*A. sterilis* Wahl No. 8	Iowa isolines X270 and X434	ASR
*Pc52*		*A. sterilis* Wahl No. 2	Iowa isoline X421	ASR
*Pc53*		*A. sterilis* 6–112-1–15	H 441	ASR
*Pc54*	*Pc35*	*A. sterilis* CAV 1832	Pendek/Pc54	ASR
*Pc55*	*Pc39*	*A. sterilis* CAV 4963	Pendek/Pc55	ASR
*Pc56*		*A. sterilis* CAV 1964	Pendek/Pc56	ASR
*Pc57*		*A. sterilis* CI 8295	H-555 or IA D640	ASR
*Pc58a*		*A. sterilis* PI 295,919	TAM-O-301	ASR
*Pc59*		*A. sterilis* PI 296,244	TAM-O-312	ASR
*Pc62*	*Pc38, Pc63*	*A. sterilis* CAV 4274	Fraser/Pc62	ASR
*Pc63*	*Pc38, Pc62*	*A. sterilis* CAV 4540	Fraser/Pc63	ASR
*Pc64*		*A. sterilis* CAV 4248	Makuru//Sun II/Pc64	ASR
*Pc67*		*A. sterilis* CAV 4656	Makuru//Sun II/Pc67	ASR
*Pc68*	Pc44, Pc46, Pc50, Pc95, PcX**	*A. sterilis* CAV 4904	Makuru//Sun II/Pc68	ASR
*Pc69*		*A. sterilis* CAV 1387	A. sterilis CAV 1387	APR
*Pc70*		*A. sterilis* PI 318,282	H547	ASR
*Pc71*		*A. sterilis* IA B437	IA Y345 or IA D526	ASR
*Pc72*		*A. sterilis* PI 298,129	IA H611-447	APR
*Pc73*		*A. sterilis* PI 309,560	IA H592	APR
*Pc74*		*A. sterilis* PI 309,560	IA H592	APR
*Pc75*		*A. sterilis* IB 2402	IB 2402/Rodney O	ASR
*Pc76*		*A. sterilis* IB 2465	IB 2465/Rodney O	ASR
*Pc77*		*A. sterilis* IB 2433	IB 2433/Rodney O	ASR
*Pc78*		*A. trichophyla* IB 1454	IB 1454/Rodney O	ASR
*Pc79*		*A. trichophyla* IB 1454	IB 1454/Rodney O	ASR
*Pc80*		*A. sterilis* IB 3432	IB 3432/Rodney O	ASR
*Pc91*		*A. magna* CI 8330	Amaglon PI497742	ASR
*Pc92*		*A. strigosa*	Obee/Midsouth PI497874	ASR
*Pc93*		*A. magna* CI 8330	*A. magna* CI8330	ASR
*Pc9*		*A. strigosa* RL1697	*A. strigosa* (RL1697)	ASR
*Pc95*	Pc44, Pc46, Pc50, Pc68, PcX**	*A. sativa* Wisc X1588-2	Jostrain	ASR
*Pc96*		*A. sativa* MG 85,039	Italia	ASR
Pc97***		*A. sterilis* CAV 1180	Harmon2/CAV1180	ASR
*Pc98*		*A. sterilis* CAV 1979	Harmon2/CAV1979	ASR
Pc100***		*A. sterilis* PI 333,699	Harmon2/PI333699	ASR
Pc101***		*A. sterilis* PI 334,961	Harmon2/PI334961	ASR
Pc102***		*A. sterilis* PI 404,589	AC Morgan/PI404589	ASR
Pc103-1***		*A. sterilis* PI 333,463	Harmon/PI 333,463	ASR
Pc104***		*A. sterilis* GS1	Morgan/GS1	ASR

*These two genes are complementary in action; both are required for resistance expression

**Also linked to the stem rust resistance genes *Pg3* and *Pg9*

***Temporary designations

In addition to designated genes, J Chong (unpublished) gave temporary designations to eight resistance genes based on rust isolate specificity and preliminary genetic analyses (viz*. Pc97-temp*, *Pc99-temp* through *Pc105-temp*; Chong J unpublished). Toporowska et al. (2021) mapped a gene present in a selection of the *Pc50* differential, given the temporary designation Pc50-5, to **chr. 6A**. Further work is needed to determine the relationships of all of these genes to those already designated.

The ability to identify and work with catalogued *Pc* resistance genes depends on the existence of single gene reference stocks for each, and isolates of *Pca* that carry matching avirulence. Although reference stocks have been nominated for all *Pc* genes designated so far, 35 are not available in single gene or single resistance specificity (in the case of complementary genes) stocks (Table 1). In these cases it is very difficult or impossible to know which of the genes present in a given stock correlates to which designated *Pc* gene. Of note in this regard is the diploid *A. strigosa* accession CI 3815, which was originally reported to carry *Pc19* (Simons et al. 1959) and *Pc30* (Marshall and Myers 1961). Subsequent studies provided evidence of five tightly linked genes, designated *Pc81–85* (Yu and Wise 2000). The genetic relationships between *Pc19*, *Pc30* and the *Pc81*–*85* complex are not known, and the designations *Pc81*-*85* were allocated without considering the potential precedence of *Pc19* and *Pc30* for at least two of these. Furthermore, unpublished studies in Australia have provided convincing evidence that CI 3815 carries *Pc94*, meaning also that the designation of *Pc94* is possibly incorrect (Park RF unpublished). Gene *Pc94* was introgressed into hexaploid oats by Aung et al. (1996) from the diploid *A. strigosa* (accession RL1697), and in later studies (J Chong unpublished), no segregation in crown rust response was observed in over 800 F_2_ plants derived from an intercross between RL1697 and CI 3815. The gene *Pc92*, present in the hexaploid lines Obee (Rothman 1984) and Obee/Midsouth (Rooney et al. 1994), is thought to have come from one of three autotetraploid *A. strigosa* accessions that included colchicine doubled Saia (*Pc15*, *Pc16*, *Pc17*) and CI 3815. Based on multipathotype tests (Park RF unpublished), gene *Pc92* is distinct from *Pc15*, *Pc16* and *Pc17* but may be present in CI 3815. The absence of single gene reference stocks for the seven genes designated from CI 3815 makes it very difficult to resolve the genetic basis of resistance to crown rust in this accession and to verify which *Pc* gene is which. A sequencing and mutational genetics approach may resolve the situation in future.

Single gene stocks have been nominated for the remaining 59 genes (this includes the complementary gene pair *Pc3*+*Pc4* in Bond), of which 12, two and three are available in NILs in the backgrounds of Pendek, Fraser and Makuru, respectively (Table 2). The genes *Pc14*, *Pc36*, *Pc50*, *Pc51*, *Pc52*, *Pc53*, *Pc57*, *Pc70*, *Pc71* and several other unknown crown rust resistance genes were also backcrossed into one or more of four backgrounds (CI 7555, CI 7970, CI 8044, Lang) by Browning and colleagues at Iowa Agriculture and Home Economics Experimental Station and USDA-ARS as part of a program to develop oat multiline cultivars (Supplementary Table S1). All of these NIL stocks have between two to six backcrosses to the recurrent parent. Because even reputedly single gene stocks may carry additional resistance genes, any assessment of virulence using these NIL stocks must always include the recurrent parent to exclude the presence of additional genes in the background.

A further complicating factor with the designation of *Pc* genes is that the allelic relationships between many are unknown. Evidence has been obtained of tight linkage or allelism between: *Pc35* and *Pc54* (McKenzie and Martens pers. comm. to Simons et al. (1978)); *Pc39* and *Pc55*; *Pc38*, *Pc62* and *Pc63*; *Pc44*, *Pc46, Pc50, Pc68, Pc95* and *PcX*; *Pc45* and *Pc53.* The latter four linkage groups are discussed in greater detail in the following section. About 60 or so of the designated *Pc* ASR genes have been shown to confer different specificities using Australian isolates of *Pca* (Park RF unpublished) and hence represent distinct resistance genes/ alleles.

Most crown rust resistance genes are dominant (e.g. *Pc68*; Wong et al. 1983), some are incompletely dominant (e.g. *Pc56*, *Pc64, Pc65* and *Pc66*), and some recessive (e.g. *Pc54* and *Pc55*). The action of some genes may be influenced by genetic background. For example, *Pc55* was recessive in crosses with Pendek (Kiehn et al. 1976), but dominant in crosses with Algerian, TAM-O-312 and a number of Pendek isolines carrying other genes (Brouwer 1983). Resistances due to complementary gene pairs were identified in cultivars Bond (*Pc3* and *Pc4,* Baker and Upadhyaya 1966), Santa Fe (*Pc7* and *Pc8*, Osler and Hayes 1953), Garry (*Pc24* and *Pc25*, Upadhyaya and Baker 1960) and Ukraine (*Pc3c* and *Pc4c*, Upadhyaya and Baker 1962). Cruz et al. (2001) identified one dominant resistance gene in UFRGS 881,920 and two complementary resistance genes to crown rust in UFRGS 15.

### Mapped catalogued ASR genes

Few of the 92 catalogued loci conferring ASR to *Pca* have been mapped to a chromosome. Some of these studies were only able to assign the genes to linkage groups that did not provide an indication of physical location in the oat genome. Mapped catalogued ASR genes were identified using a number of different molecular methods including: SCAR (Sequence Characterised Amplified region) markers adapted from probes developed for other cereals; RFLP (Restriction Fragment Length Polymorphism) makers based on short fragments of digested DNA; RAPD (Random Amplified Polymorphic DNA) fragments from PCR amplification of random segments of genomic DNA with single primer of arbitrary nucleotide sequences; AFLP (amplified fragment length polymorphism) based on the detection of genomic restriction fragments by PCR amplification; DArT (Diversity Arrays Technology) marker microarray hybridizations based on restriction enzymes; and cDNA-derived single-nucleotide polymorphism (SNP) markers. The genes for which mapping and/or marker development have been undertaken are detailed as follows:

*Pc38, Pc62* and *Pc63* Harder et al. (1980) conducted genetic analyses of these three genes and found that they were either linked or allelic. Wight et al. (2004) mapped *Pc38* to a region homologous to KO (Kanota/ Ogle) group 17 (Mrg02, **[chr. 7D]**) in two F_2:3_ populations.

*Pc39*, *Pc55*, and *Pc71* Kiehn et al. (1976) found that *Pc39* and *Pc55* are either very closely linked or allelic. Based on very similar responses of differentials carrying *Pc39*, *Pc55,* or *Pc71* to 130 isolates of *Pca* collected off *A. sterilis* and *A. barbata* in Israel and 683 isolates from *A. sativa* and 73 aecial collections from buckthorn in the USA, Leonard et al. (2005) concluded that these genes are either identical or allelic. Chong and Seaman (1989) similarly reported complete association between virulence for *Pc39* and *Pc55* in Canada. This is consistent with results from surveys of virulence in *Pca* in Australia, where identical responses were documented for differentials carrying each of the three resistance genes in tests of over 1,400 isolates of *Pca* collected between 2000 and 2020, and 152 historical isolates dating back to the 1950s (Park RF, unpublished).

Wight et al. (2004) mapped *Pc39* to a region homologous to KO group 37. Two more recent studies mapped *Pc39* to Mrg11 **[chr. 4C]**. Sowa and Paczos-Grzeda (2020) mapped *Pc39* to Mrg11. Six tightly linked PCR-based SCAR markers were validated on a set of 22 oat cultivars, confirming the presence of *Pc39* in 16 of these (e.g. cvv. Celer (*Pc39*), AC Assiniboia (*Pc38*, *Pc39*, *Pc68*), AC Medallion (*Pc38*, *Pc39*, *Pc68*), Riel (*Pc38*, *Pc39*), Stainless (*Pc38*, *Pc39*, *Pc68, Pc91*)), and its absence in three (Bingo, Borys and Dragon). Zhao et al. (2020a) again mapped *Pc39* to Mrg11 [**chr. 4C**]. Kompetitive Allele-Specific PCR assays for two *Pc39*-linked SNP loci accurately predicted *Pc39* in two genotypes, and its absence in 72 diverse oat genotypes.

Bush and Wise (1998) used a high density RFLP map to localise *Pc71* to a group of 12 linked RFLP markers within 6 cM either proximal or distal to the gene. All markers were positioned in the centre of linkage group 11 of the Kanota/Ogle map, which Sowa and Paczos-Grzeda (2020) attributed to Mrg21 **[chr. 4D]**. Esvelt Klos et al. (2017) mapped *Pc71* to Mrg05 **[chr. 6A]**. Should *Pc71* be located on **chr. 4D** or **chr. 6A**, it cannot be allelic with *Pc55*. If these genes prove to be genetically independent, they do nonetheless confer the same resistance specificity and are of equal value in resistance breeding. Clearly, more work is needed to resolve the positions and genetic relationships between genes *Pc39*, *Pc55* and *Pc71*.

*Pc45* and *Pc53* Kebede et al. (2019) examined the genetic relationship between a tentatively designated crown rust resistance gene *PcKM*, previously found and mapped in the oat cultivars Kame and AC Morton (Gnanesh et al. 2015) and *Pc45*. They mapped both resistances across five populations and localised both on Mrg08 **[chr. 2D]**, concluding that they were one and the same. They identified two codominat KASP markers as being the most accurate, predicting the presence of *Pc45* and *PcKM* in two oat genotypes, and their absence in 74 diverse oat genotypes. Esvelt Klos et al. (2017) also mapped *PcKM* to Mrg08 **[chr. 2D]**.

Admassu-Yimer et al. (2018a) demonstrated close linkage between *Pc45* and *Pc53* of within 1 cM on Mrg08 **[chr. 2D]**, suggesting they are either tightly linked or allelic.

*Pc44*, *Pc46*, *Pc50*, *Pc68*, *Pc95*, and *PcX* A series of studies that led to the designation of these genes provided evidence that they form a large cluster along with the stem rust resistance genes *Pg3* and *Pg9*. Initial studies by McKenzie and Green (1965) demonstrated repulsion linkage or allelism between *Pg3* (gene “E”) and *Pg9* (gene “H”), and subsequent studies showed linkage between *Pg9* and *Pc44* (Martens et al. 1968), *Pg9* and *PcX* (Chong et al. 1994), and *Pg3* and *Pc95* (Harder et al. 1995). Chong et al. (1994) also established linkage between *Pg9*, *PcX* and *Pc68*, Wong et al. (1983) between *Pc68* and *Pc46*, and Fleischmann et al. (1971) between *Pc46* and *Pc50*, indicating that *Pc46*, *Pc50* and *Pc68* are also part of this linkage group. Chong et al. (1994) considered *PcX* to be either *Pc6c* or *Pc9*. Given that both the *Pc6* and *Pc9* loci comprise multiple alleles (Tables 1 and 2), either would add to the complexity of this resistance gene cluster.

Based on RFLP probes that were previously shown to be closely linked to *Pg9*, Chen et al. (2006) developed two SNP markers and an STS marker that were linked with *Pc68* at recombinational distances of 4.2 cM and 6.7 cM in two populations. Based on the original RFLP markers, *Pg9* (and hence *Pc68*) were located to the Kanota/Ogle linkage group 4_12 (spread across Mrg19 and 23; **chr. 3D** and **chr. 3A**, respectively). As found by Chen et al. (2006), Kulchelski et al. (2010) mapped *Pc68* to linkage group 4_12 of the Kanota/Ogle linkage map. The GWAS study of Esvelt Klos et al. (2017) confimed the location of *Pc68* on Mrg19 **[chr. 3D]**, and also identified a SNP marker that could be suitable for MAS of this gene.

*Pc48* Wight et al. (2004) mapped *Pc48* to a region corresponding to the KO group 22_44 + 18 based on linakge with five RFLP markers (Mrg20 **[chr. 4A]**). Interestingly, they found differences in marker linkage in two populations based on the resistant parent Pendek-48. Further genotyping of different plants within Pendek-48 showed that it comprised at least two genotypes carrying different sized introgressions with *Pc48* from *A. sterilis*. GWAS studies by Esvelt Klos et al. (2017) confirmed the location of *Pc48* on Mrg20 **[chr. 4A]**.

*Pc54* Admassu-Yimer et al. (2022) mapped *Pc54* to Mrg02 **[chr. 7D]** in two biparental RIL populations. The location of 60–84 cM on Mrg02 was considered by Adamssu-Yimer et al. (2022) as possibly overlapping with that reported for Pc38 by Wight et al. (2004).

*Pc58* Hoffman et al. (2006) demonstrated the presence of three ASR genes in TAM-O-301 in crown rust tests of an Ogle/TAM-O-301 F_6:7_ RIL population. Because the resistance in TAM-O-301 had been previously designated *Pc58*, the authors designated these genes *Pc58a*, *Pc58b*, *Pc58c*. In wheat, such designations would indicate allelism. The three loci were however mapped to a region spanning 41.0 cM as defined by RFLP markers, with gene *Pc58a* and *Pc58c* being closely linked and *Pc58b* being independent of these two loci. Overall, the three genes were mapped to two previously described linkage groups, OT32 and OT33, both of which correspond to KO linkage group 17 (hence Mrg02 **[chr. 7D]**). The **chr. 7D** location was confirmed in the GWAS analysis by Esvelt Klos et al. (2017).

Jackson et al. (2008) used the Ogle/TAM-O-301 F_6:7_ RIL population to map ASR to two pathotypes of *Pca* that were virulent on Ogle but avirulent on TAM-O-301. They mapped a single gene that conferred a “bleached fleck” phenotype, different to that conferred by *Pc58*, to both pathotypes to the linkage group OT-11 (homologous to KO_4). Detailed measurements of uredinial length (UL) identified major QTL on linkage groups OT-11 and OT-32. The latter was mapped very closely (0.3 cM) to the previously determined location for *Pc58a*. The same pathotypes were used to phenotype the population in the field at adult plant growth stages for infection type, UL and relative fungal DNA (FDNA). Measurements of FDNA in both greenhouse and field experiments similarly mapped OT-32 very close to *Pc58a*. It was concluded that the reductions in UL and FDNA were due to either *Pc58a* or genes tightly linked to this locus. Three minor QTL were also detected, on linkage groups OT-8 and OT-15 (from TAM-O-301) and OT-27 (from Ogle).

Jackson et al. (2007) also investigated resistance to *Pca* in Ogle in the Ogle/TAM-O-301 F_6:7_ RIL population using an isolate of *Pca* that was avirulent on Ogle and virulent on TAM-O-301. Based on greenhouse assessments and artificially inoculated field plots, a single gene for resistance derived from Ogle was mapped to linkage group OT6 (homologous to linkage group KO). A previous study by Bush and Wise (1996) mapped two unlinked resistance genes derived from Ogle to the linkage groups KO_4 and KO_13. Based on pedigree analysis, Jackson et al. (2007) concluded that the gene detected in their study was likely derived from either Landhafer (*Pc4*, *Pc5*) or Victoria (*Pc2*, *Pc11*, *Pc12*).

*Pc91* Using aneuploid stocks, Rooney et al. (1994) localised *Pc91* to chromosome 18. The gene was further mapped by McCartney et al. (2011), who developed five robust PCR-based SCAR markers that were validated on 23 North American oat cultivars/ breeding lines to confirm its presence in cultivars HiFi, Souris and Stainless carry *Pc91*, and absence in Ogle, Leggett, Ronald CDC Sol-Fi, CDC Minstrel, CDC Dancer and CDC Weaver. Aligning the linkage map generated with the Kanota/Ogle linkage maps indicated that *Pc91* was likely located on either chromosome 7C **[chr. 1C]** or 17A **[chr. 1A]**.

In a subsequent study, Gnanesh et al. (2013) developed three KASP assays that accurately predicted the presence/ absence of *Pc91*. Further mapping located *Pc91* to the intergenomic translocation involving chromosomes 7C and 17A that is present in most spring oat genotypes.

Esvelt Klos et al. (2017) mapped *Pc91* to Mrg18 **[chr 1A]** and this was further validated by Maughan et al. (2019)

*Pc92* This gene was identified by Rooney et al. (1994) in the hexaploid oat breeding line Obee/Midsouth. Using backcross-derived lines, RFLP markers linked to *Pc92* were identified. The locus was then mapped using segregating F_2_ populations, and an attempt to determine chromosomal location using aneuploid stocks was unsuccessful presumably because of the lack of the corresponding aneuploid stock.

*Pc94* Chong et al. (2004) and Chen et al. (2007) undertook genetic studies of *Pc94* and although they did not map the gene they did develop linked markers for use in resistance breeding.

*Pc98* Zhao et al. (2020b) mapped *Pc98* in two F_2:3_ populations based on genotypic data generated with the 6 K Infinium iSelect SNP array, and using the consensus map developed by Chaffin et al. (2016) and Bekele et al. (2018), mapped *Pc98* to Mrg20 **[chr. 4A]**.

To further identify probable differences from the current consensus map that may be attributable to segregating translocations and to map genes or quantitative trait loci (QTL) affecting oat crown rust resistance, Sunstrum et al. (2019) developed a de novo genetic linkage map in a population based on a breeding line from southern USA (TX07CS-1948) and a Canadian spring oat line (SA04213) using high‐density GBS markers. This linkage map served as a useful tool in detecting four QTL associated with resistance to *Pca*, of which two (on linkage groups Mrg02 and Mrg19) corresponded with QTL detected in a previous GWAS by Esvelt Klos et al. (2017). These QTL can be reconciled to known *Pc* genes loci or resistance gene clusters including *Pc38* (Wight et al. 2004) and *Pc58a* (Hoffman et al. 2006; Jackson et al. 2007) on Mrg02, and *Pc68* (Kulcheski et al. 2010) on Mrg19. The collation of marker details can be used as a tool to assist in reconciling the most probable location for a given gene. Marker sequences of probes associated with the mapped catalogued ASR genes detailed above were retrieved from the GrainGenes database (wheat.pw.usda.gov; Blake et al. (2019)) and the inbuilt Basic Local Alignment Search Tool (BLAST) used to assign physical positions in chromosomes of the PepsiCo OT3098 Hexaploid Oat v2 pseudomolecules (2021) (Table 3 as Electronic supplementary material). Although the genome assembly is advanced, it is still being resolved and functional homologs have been detected. Due to the short read length of probes reported in previous studies and the highly repetitive nature of the oat genomes, the presence of multiple complementary sequences with reported markers is expected. Therefore, the top three the BLAST hits with E values below 10^–5^ are presented for each marker. Descriptions of the markers linked to *Pc* genes, mapping populations and sequence accession are also presented in Table 3 (as Electronic supplementary material).

### Mapping studies of uncharacterised “field” resistance to *Pca* at adult plant growth stages

QTL mapping and GWAS approaches have been used to map resistance to rust in wheat, barley and oat based on rust response data from field plots. The resistance observed in many of these studies is referred to as “field” resistance as it is effective at adult plant growth stages under field conditions. In most cases, the rust pathotype(s) present in the field plots were however unknown, meaning that it is impossible to attribute the field resistance documented to either ASR, APR, or both.

The crown rust resistance of the hexaploid line MAM17-5, believed to carry two dominant resistance genes to *Pca* derived from one or both of two *A. strigosa* accessions (viz. CI 3436 and CI 3820), was studied in an F_5:6_ RIL population based on the cross MAM17-5/ Ogle (Zhu and Kaeppler 2003). The population was assessed under field conditions in Wisconsin across two years, using natural and artificial inoculation of a composite mixture of *Pca* pathotypes common to the Midwest of the USA at the time. Two QTL, referred to by the authors as “field” resistance, were detected and mapped to linkage groups but with no reference to other consensus maps. In subsequent studies of the same population, Zhu et al. (2003) mapped ASR resistance to three North American pathotypes of *Pca*. Combined, these two studies confirmed the presence of two QTL, one of which (*Pcq1*) mainly controlled field resistance and one of which (*Pcq2*) mainly controlled greenhouse ASR. Given that the resistance examined in these two studies was derived from *A. strigosa* accession CI 3436 or CI 3820, and that the ASR gene *Pc23* was described from *A. strigosa* accession CD3820 (Table 2), it could be that at least one of the loci mapped is in fact *Pc23*.

Barbosa et al. (2006) characterised resistance to *Pca* in the oat genotype UFRGS910906, which the authors stated carried partial resistance to *Pca*. UFRGS910906 was crossed to the *Pca* susceptible UFRGS7 and assessed under field conditions with natural infection at the F_2_ in 1999 and at the F_6_ in 2000. Five QTL were detected at the F_2_, none of which were detected at F_6_, in which three different QTL were found. The simplest explanation for these results is that different *Pca* pathotypes prevailed in each year. Given that the identity of the pathotypes present were unknown, and that the ASR status of both parents is unknown, it is impossible to attribute the QTL detected to either ASR or APR.

Jackson et al. (2007) evaluated the ability of three different assessment methods (quantitative real-time q-PCR to estimate fungal growth in the host, digital image analysis, and visual ratings) to map crown rust resistance in cvv. Ogle and TAM O-301. A major gene in Ogle was identified and mapped by all three assessment methods to linkage group OT6 although the resolution produced by q-PCR enabled more precise mapping. Data generated by the latter method permitted identification of QTL on linkage groups OT32 and OT2.

The field resistances of CDC Boyer and the oat breeding line 9417A1-9–2-2–2-5 were studied by Babiker et al. (2015) in three F_6:8_ RIL populations across two field sites (Louisiana State University Baton Rouge (2 years) and the Cereal Disease Laboratory (CDL) St Paul, MN (3 years)). The authors refer to the resistance assessed as “partial” resistance, but it is impossible to know if it was ASR or APR in the absence of information of the pathotypes present. Four QTLs were detected: three from CDC Boyer (two on chromosome 19A [**chr. 4A]** and one on 12D [**chr. 2D**]), and one from 9417A1-9–2-2–2-5 (on 13A [**chr. 7A**]). Only one of these, *Qcr.cdl9lsu9-19A,* was detected at more than one field site. To date, two ASR genes have been mapped to **chr. 4A** (viz. *Pc48* and *Pc98*), and two to **chr. 2D** (*Pc45* and *Pc53*). In the absence of information regarding the virulence of the pathotypes present in the nurseries, it is not possible to discount one or more of these gene as contributing to the resistances identified.

Montilla-Bascón et al. (2015) used a GWAS approach to map ASR resistance in 177 oat accessions to a single pathotype of *Pca* with broad virulence collected from Spain. Positive associations with five markers were found, two of which were located on KO linkage groups 32 **[chr. 5C]** and 17 **[chr. 7D]**. The identities of these resistances are unknown; *Pc38* and *Pc58* have been mapped to **chr. 7D**, however the genes detected cannot be these because the pathotype used was virulent for both.

A set of 1,000 *A. sativa* genotypes collected prior to 1930 and landraces collected in the 19^th^ and 20^th^ Centuries was assessed for crown rust response in the CDL St Paul Buckthorn nursery in 1994 and 1997 (Winkler et al. 2016). The response data were not consistent across the years, but singly provided evidence of associations with five linkage groups, two of which could be associated with catalogued *Pc* genes of known map location (viz*.* Mrg08 (possibly *PcKM*/*Pc45*), Mrg28 (possibly *Pc91*), Mrg20 (possibly *Pc48*)), and two on Mrg23 and Mrg01 that were of unknown identity. All loci had closely linked SNPs.

Esvelt Klos et al. (2017) used a GWAS approach to map ASR and field resistance to *Pca* in a set of 631 oat lines representing modern elite germplasm and 31 *Pca* differential genotypes. All were genotyped using the Illumina oat iSelect 6 K beadchip array, tested as seedlings for response to 10 pathotypes of *Pca*, and field tested as adult plants in 10 location/ years in the USA and Canada across six field sites (2010 and 2011: Castroville Texas, Fargo North Dakota, St Paul Minnesota (in the CDL buckthorn nursery), Winnipeg Manitoba; 2011 only: Baton Rouge Louisiana, Ottawa Ontario). A total of 29 SNPs in 12 linkage groups were predictive of crown rust response across seedling and adult plant tests. The authors conceded that interpreting these associations with respect to the type of resistance (ASR vs APR) and identity with respect to previously designated *Pc* genes was not simple. Analyses of the 31 *Pca* differential genotypes did not provide useful information in relating the QTL detected in the 631 oat lines to catalogued *Pc* genes. Ten SNPs were primarily associated with seedling response and were regarded as ASR genes that are no longer effective in the field in North America due to matching virulence. Six SNPs were exclusively associated with adult plant field response to crown rust; however, none were detected at all year/ locations: one field site (1 QTL); two year/locations (3 QTL); five year/ locations (1 QTL on Mrg18 at 68 cM **[chr. 1A]**); 8 year/ locations (1 QTL on Mrg06 at 74 cM **[chr. 5D]**). The remaining 13 SNPs were associated with both seeding and adult plant response, and it was suggested that these could represent ASR genes that were effective at some field sites in some years.

### Genetic studies of adult plant resistance to *Pca* in oat

Much less is known about the genetic basis of APR to rust pathogens in cereals compared to ASR. For example, while it is known that many Australian wheat cultivars carry APR to stripe rust, only two of the genes responsible for this can be reliably identified (viz. *Yr18* and *Yr29*; Park et al. 2020). The principal reason behind this is that APR is technically more difficult to work with than ASR: phenotyping must be undertaken on adult plants and is only reliable if it is conducted in field nurseries that have been artificially inoculated with well-defined rust pathotypes that are virulent for any ASR genes present in the material being assessed. Added complications are that the resistance conferred by APR is often intermediate in effect, the onset of expression of different APR varies with growth stage, and the level of expression can be affected by inoculum load and environment.

#### Catalogued genes conferring APR

Of the 92 loci conferring resistance to *Pca* in *Avena* that have been catalogued, six confer APR. The chromosomal locations of these genes are unknown.

Studies conducted at the University of Sydney by Upadhyaya and Baker (1960) found that the oat cultivars Victoria and Garry (a Victoria derivative) carried the same resistance genes. Two complementary genes conferred seedling resistance to *Pca* (*Pc24* + *Pc25*), but these genes were not effective at adult plant growth stages. APR to *Pca* in Garry was attributed to two genes that were designated *Pc27* and *Pc28*. A third gene conferring ASR in Garry, *Pc26*, was later reported by Upadhyaya and Baker (1962). Field tests of the adult plant crown rust response of Garry at the Plant Breeding Institute Cobbitty have shown that it is quite resistant (10MR), despite being susceptible in greenhouse seedling tests (Roake J and Park RF, unpublished). Further genetic studies are required to determine the inheritance of this resistance.

Harder et al. (1984) reported APR to crown rust in *A. sterilis* accession CAV 1387. The inheritance of the resistance was studied by crossing CAV 1387 with the *A. sativa* cultivar Fraser, and shown to be governed by a partially dominant gene designated *Pc69*. Further rust testing of a putative single gene line derived from CAV 1387 showed heterogeneity among plants at both seedling and adult plant growth stages, and did not detect APR (Chong J, unpublished).

Three genes from *A. sterilis* were reported by Simons and Michel (personal communication to https://www.ars.usda.gov/midwest-area/stpaul/cereal-disease-lab/docs/cereal-rusts/oat-crown-rust/unpublished) as conferring APR to crown rust: *Pc72* from *A. sterilis* accession PI 298,129. *Pc73* and *Pc74* from *A. sterilis* accession PI 309,560, tester line being IA H592. The level of protection afforded by these resistances has not been assessed critically; accessions believed to carry *Pc72* or *Pc73* were completely susceptible in the field in Poland (Paczos-Grzeda E and Sowa S, unpublished), and line IA H611-447 (*Pc72*) was rated as 5S under field conditions in Australia (Roake J and Park RF, unpublished).

#### Uncatalogued genes conferring APR

Several oat cultivars or accessions have been reported to carry uncharacterised APR to crown rust; however, the genetic basis of the resistance in these is largely unknown. APR to crown rust was reported in the cultivars Santa Fe and Ukraine by Upadhyaya and Baker (1965); however, the genetic basis of these resistances was not studied and it is unknown if it is distinct from the APR in Garry reported previously by these authors. Both cutivars also carry designated ASR genes (*Pc6*, *Pc7*, *Pc8* and *Pc21* in Santa Fe; *Pc3c*, *Pc4c*, *Pc6c* and *Pc9* in Ukraine). Red Rustproof oats were introduced into southern USA in the mid 1800s, from which improved “strains” were selected (Coffman et al. 1961). Luke et al. (1972) found that the slow rusting of Red Rustproof 14 (CI 4876), a selection made from Red Rustproof around 1865, was stable over an 11 year period, and later showed high broad-sense heritability and was governed by an estimated 2.16 genes (Luke et al. 1975). The cultivars Ajax, Beaver and Erban (Welsh et al. 1953), and Lodi, Portage and Rodney (Heagle and Moore 1970), were also reported to carry uncharacterised APR to *Pca*. By using specific pathotypes of *Pca* in greenhouse seedling and adult plant field tests, Simons (1961) identified three “strains” with high levels of APR (viz*.* PI 174,545, PI 185,783, and PI 197,278). While all three have been assessed under Australian conditions, only one has shown good levels of APR (PI 174,545) (Roake J and Park RF, unpublished). In a subsequent study, Simons (1975) undertook genetic studies of the APR in two of these lines (viz. PI 174,545 and PI 185,783) and two others that were found in the 1961 study to have low levels of APR (viz. PI 174,544 and PI 19,727), and found continuous variation in response that was suggestive of polygenic inheritance.

Briére et al. (1994) assessed the adult plant field responses of 33 oat genotypes over two years. The plots were inoculated with a pathotype of *Pca* that developed sporulating pustules on all genotypes under controlled environmental conditions. Two breeding lines (OA 712-17 and OA 712-33) and four cultivars (Glen, Woodstock, Sylvya, Fidler) had high levels of what the authors termed partial resistance. Genetic studies have shown that both Fidler and Woodstock carry *Pc39* (Chong J, unpublished), and it is unclear whether this ASR gene was effective or not in the studies by Briére et al. (1994).

As mentioned previously, Jackson et al. (2008) mapped resistance to *Pca* in TAM-O-301 based on an F_6:7_ RIL population (Ogle/TAM-O-301). Seedling tests with two pathotypes of *Pca* identified two ASR genes, one of which was considered to be *Pc58* (“*Pc58a*”). Field tests at adult plant growth stages using the same two pathotypes allowed the identification of two minor QTL from TAM-O-301 and one from Ogle.

The USDA Cereal Disease Laboratory (CDL) at St Paul Minnesota established a buckthorn (*Rhamnus carthartica*) nursery in 1953. Named after the pathologist who established it, the Matt Moore Buckthorn Nursery comprises hedges of *R. carthartica* between which plots of oat can be sown and assessed for response to a multitude of pathotypes of *Pca* arising from sexual recombination on its aecial host. The nursery was used by Stuthman and Moore (1970) and coworkers to select crown rust resistant lines from breeding populations based on crosses designed to combine different types of resistance from diverse sources. Leonard (2002) undertook detailed greenhouse (seedling) and field (adult plant) studies of the crown rust resistance in 14 of the lines produced by Stuthman and Moore that were resistant in the buckthorn nursery in annual tests over an 8 year period. The cultivar Portage, known to carry moderate levels of slow rusting to *Pca*, was included as a control. Portage also demonstrates slow rusting under Australian conditions (Roake J and Park RF, unpublished). All lines were resistant to crown rust across field tests over seven years, many with resistance superior to that of Portage. Although greenhouse studies indicated that at least some of the resistance observed was likely due to ASR, four lines were identifed with APR, two of which had levels of APR adequate enough to protect against *Pca* in the absence of ASR (viz. MN841801 (pedigree: Florad/Coker 58–7/3/CI7558//Black Mesdag/Aberdeen 101 [65B663/65B1362]; MN841804 (pedigree: CI7683//Black Mesdag/Aberdeen 101/3/Rodney/4/CI7558//Black Mesdag/Aberdeen 101 [65B1286/65B1362]). Given the similarity in the pedigrees of these lines, there may be some commonality in the genes underlying the APR they carry. Both lines have displayed good levels of APR to crown rust under Australian field conditions in artificially inoculated nurseries over at least 5 years (Roake J and Park RF unpublished). Several studies have been conducted to investigate the genetic basis of APR in MN841801, these are described below.

Chong (2000) conducted crown rust tests of adult plants of an F_7:9_ RIL population based on MN841801 in a growth chamber to show that this line carried two complementary (or additive) genes for APR. Lin et al. (2014) phenotyped the same RIL population and two others based on MN841801 for adult plant response to crown rust in the field over nine site/years. All plots were inoculated with the same pathotype used previously in the growth chamber study (Chong 2000). A major effect QTL (RMR to MR/MS), *QPc.crc-14D*, was detected in all three populations in all environments. *QPc.crc-14D* was mapped to chromosome 14 D **[chr. 5D]**. Two KASP markers were identified that provided strong evidence for the presence of *QPc.crc-14D* in three oat genotypes (viz. 02P07-BC1A, W020203 and Clav 8361). Comparative mapping suggested that *Qpc.crc-14D* is homologous to the wheat stripe rust resistance gene *Yr16*, which was mapped in two independent studies to wheat chromosome 2D (Worland and Law 1986; Agenbeg et al. 2012). Additional seedling tests of the RILs of the three populations with pathotypes virulent on AC Assiniboia, AC Medallion and Makuru showed the MN841801 parent was heterogeneous for ASR, carrrying either two genes or one gene depending on the RIL population used (J. Chong and J. Lin, unpublished).

Portyanko et al. (2005) tested an F_6:8_ RIL population based on the crown rust susceptible genotype Noble-2 and MN841801-1 in the field twice at St Paul Minnesota (1997 and 1998; plots inoculated with a bulk population of *Pca*) and once at Rosemount Minnesota (1998, natural inoculum), and under greenhouse conditions as adult plants using a *Pca* pathotype that was virulent on both parents at seedling growth stages. A total of seven QTL were detected, accounting for up to half the variation associated with resistance of MN84801-1, four of which were considered major QTL of which one (*Prq2* on linkage group 26) had the most consistent effect. A lack of common markers across different studies made comparisons difficult, but the QTL *Prq1b* was considered to be associated with *Pc38*/ *Pc62*/ *Pc63* (**[chr. 7D]**).

The population developed by Portyanko et al. (2005) was advanced to F_6:9_ by Acevedo et al. (2010) and phenotyped for adult plant response to *Pca* over six sites/ years and in the greenhouse. Three field sites and the greenhouse tests used two pathotypes of *Pca* that were virulent on seedlings of MN841801-1 and Noble-2, one of which was the same pathotype used by Chong (2000) and Lin et al. (2014). Overall, the study detected the same seven QTL as reported by Portyanko et al. (2005), plus an additional one (*Prq8*) that was detected at two field sites only. It was concluded that four QTL (viz. *Prq1a*, *Prq2*, *Prq7*, *Prq8*) were responsible for the partial resistance of MN841801-1 to crown rust. As was found by Portyanko et al. (2005), *Prq2* had the most consistent effect; this along with *Prq1a* accounted for 30.1% of the variation associated with the observed resistance to *Pca*. The region containing QTL *Prq1a* (linkage group MN3) is homologous to the linkage group KO-17 (Mrg02 **[chr. 7D]**).

Differences in findings from the above studies of the resistance of MN841801 clearly demonstrate the difficulties of genetic studies of rust resistance, especially APR. The number of genomic regions associated with APR to *Pca* in these studies varied from one (Lin et al. 2014), to two (Chong 2000), to four (Portyanko et al. 2005; Acevedo et al. 2010). The lack of congruity between the above APR studies could relate to genetic heterogeneity in the original accession of MN841801, pathogen virulence, the different methods (e.g. growth chamber vs field) used to assess crown rust response, and/or environment. Of particular significance among these is pathogen virulence, as using the correct rust isolates is the only way APR can be distinguished from ASR. The MN841801 line used as parent by Chong (2000) and Lin et al. (2014) was shown to be heterogeneous for ASR. The discrepancy in findings between the studies by Chong (2000) and Lin et al. (2014) could be due to the different methods used. The detection of two additive APR genes by Chong (2000) was based on a single inoculation in growth chamber experiments. Rust reactions from a single inoculation would be useful for identification of infection types but would not be useful for detecting resistance that expresses quantitatively over multiple disease cycles in field experiments. Alternatively, it is possible that a second APR QTL was not detected by Lin et al. (2014) because their linkage map was incomplete. Differences in findings between the studies by Lin et al. (2014) and Acevedo et al. (2010) are more difficult to explain because the same pathotype of *Pca* was used in both studies.

The Matt Moore Buckthorn Nursery was also used by Admassu-Yimer et al. (2018b), who undertook adult plant tests on 607 *A. sativa* accessions from 44 countries in the nursery and at Baton Rouge Louisiana. A total of 36 genotypes were identified that were resistant at both field sites, and these were tested a second time at both field sites. Seedling tests of the 36 genotypes identified three that were seedling susceptible to all eight pathotypes used. These three genotypes were tested for adult plant response to each of the eight pathotypes in growth cabinets in two experiments, and ranged in response from 5 to 30MR (Clav 3390), from 5MR to 40MS (Clav 2272), and from TrMR to 30MS (PI 285,583), confirming the presence of good levels of APR in all. The genetic basis of the resistance in the lines is unknown as no genetic analyses were undertaken.

All seven designated *Pc* genes conferring APR to *Pca* are from the hexaploid species *A. sativa* (*Pc27* and *Pc28*) and *A. sterilis* (*Pc69*, *Pc72*, *Pc73*, *Pc74*). Using controlled inoculations of seedling and adult plants with specific pathotypes, Cabral et al. (2011) identified six diploid *A. strigosa* accessions (CIav6956, CIav7280, CIav8089, CIav9020, PI292226, PI436082) and one tetraploid *A. barbata* accession (PI337865) that carried APR to *Pca*. The APR of three diploid accessions of diverse geographic origins (Canada, Chile, USA) was shown to be governed by single dominant genes, the genetic relationship between which was not investigated. Although being expressed at adult plant growth stages only, the three genes conferred a hypersensitive phenotype typical of many non-durable ASR genes. This could suggest a lack of durability of these APR genes, as was found in wheat with the hypersensitive APR gene *Lr12* (Park and McIntosh 1994).

## Breeding for resistance to *P. coronata* f. sp. *avenae*

### Early efforts

Simons (1970, 1985) and Coffman et al. (1961) provided comprehensive summaries of the early use of resistance to control *Pca* in oat in North America. Two important cultivars in this were Victoria and Bond. The latter was developed in Australia from a cross between the Swedish cultivar Golden Rain and a selection from the genotype Red Algerian (Baker 1966). Baker and Upadhyaya (1966) and Baker (1966) identified two complementary resistance genes in Bond, later designated *Pc3* and *Pc4*. Although virulence to Bond was first detected in Australia in 1948 by Waterhouse (1952), the frequency of virulence remained low for some time after that and the resistance remained useful (Baker and Upadhyaya 1966). Virulence to the Bond resistance became common in the USA along with the adoption of Bond-derived cultivars (e.g. Bonda, Andrew, Camelia; Simons 1970), to the point where by 1953 this resistance was of almost no value at all (Simons 1970). The cultivar Victoria was introduced to North America from South America in 1927. North American studies of the genetic basis of resistance to *Pca* in Victoria reported the presence of one (Murphy et al. 1937; designated *Pc2*) or two (Chang and Sadanga 1964; *Pc2* and *Pc11*) resistance genes, of which *Pc2* was used in developing many oat cultivars released during the 1940s (e.g. Tama, Vicland, Vikota, Cedar; Coffman et al. 1961), which later succumbed to Victoria blight caused by *Helminothosporium victoriae* because of the association between *Pc2* and susceptiblility to this pathogen caused by the pleiotropic or tightly linked locus *Hv-1*. The spread of Victoria blight was rapid and losses were estimated in the order of hundreds of millions of dollars (Coffman et al. 1961).

The cultivars Bond and Victoria were the sole sources of resistance to *Pca* used in Australia prior to 1960 (Upadhyaya and Baker 1960). The development of virulence for both cultivars there and the emergence of *Pca* race 45 in the USA, which rendered Bond and its derivatives susceptible and caused high losses in southern USA (Coffman et al. 1961), led to searches for new resistance to *Pca*. Studies of the genetic basis of resistance to *Pca* in Bond, Victoria, Garry (a Victoria derivative), Landhafer (originating from Germany), Santa Fe (Argentina), Trispernia (Romania) and Ukraine (Russia) (Upadhyaya and Baker 1960, 1962, 1965; Baker and Upadhyaya 1966, Baker 1966) demonstrated considerable genetic diversity in both ASR and APR to *Pca*. Landhafer, Santa Fe and Trispernia were used as sources of crown rust resistance in North America in the 1950s (e.g. Clintafe, Seminole, Fayette, AB 110 from Santa Fe; Floriland, Sunland, Clintland, Minhafer, Radar 1 and Radar 2, from Landhafer; Moregrain, Suregrain from Trispernia; Coffman et al. 1961). Two varieties based on Landhafer were released in Australia (Minhafer, Stout; Fitzsimmons et al. 1983). Pathogenicity surveys in the USA by Simons and Murphy (1955) detected virulence on Landhafer, Santa Fe and Trispernia. Based on pathogenicity surveys in Australia between 1952 and 1954 in which virulence for the ASR in all of these oat genotypes and Klein 69B (*Pc10*) was detected, Baker and Upadhyaya (1955) stated that the “situation as regards the future of resistance breeding is not particularly straightforward”, and recommended assessing the value of combining different resistances to reduce the probability of the pathogen acquiring virulence, mutation to induce new resistance specificities, and wide crossing to introgress new resistance.

### Alien species as sources of new resistance to crown rust

Of the 92 designated loci conferring resistance to *Pca*, only 25 come from the hexaploid species *A. sativa* and *A. byzantina,* highlighting the importance of other *Avena* species as sources of resistance (Tables 1 and 2). During the 1960s, attention turned to the wild hexaploid species *A. sterilis* as a source of resistance to *Pca*, with most of the genes designated after *Pc34* originating from this species (Table 2). Although 19 loci have been designated from the diploid *A. strigosa*, only three of these, *Pc23* (Dyck and Zillinsky 1963), *Pc92* (Rooney et al. 1994) and *Pc94* (Aung et al. 1996) are available in stable hexaploid lines. The breeding line WIX4361-9 (“WIX”) was shown by Bonnett (1996) to carry two resistance genes. Based on rust isolate specificity and pedigree (WIX has the pedigree N569-42–51/Froker/2/Froker/WIX1274-2, with N569-42–51 being an accession of *A. strigosa*), one of these two genes is likely *Pc15*, *Pc16* or *Pc17* derived from Saia, and the second *Pc39* (Park RF unpublished). Gene *Pc91* originated from the tetraploid species *A. magna* (Rooney et al. 1994). *A. magna* accession CI 8330 was crossed by the diploid *A. longiglumis* (accession CW 57), and after treating with colchicine a single F_1_ seed was obtained from which the synthetic hexaploid line Amagalon was generated. The *A. magna* accession CI 8330 is also listed as the source of a second gene, *Pc93*, (https://www.ars.usda.gov/midwest-area/stpaul/cereal-disease-lab/docs/resistance-genes/resistance-genes/), however no further details are available concerning this resistance gene.

### Deployment of *Pc* genes and the emergence of matching virulence

The crown rust resistances of Victoria, Bond, Landhafer, Santa Fe, and Trispernia provided transient, short termed resistance only in both North America and Australia, with the development of matching virulence in *Pca* following soon after cultivar release and often significant losses resulting. The deployment of many different ASR genes since then has similarly met with the rapid appearance of matching virulence in *Pca* populations; Carson (2009) for example reported that oat cultivars with major gene resistance to crown rust in the USA were generally rendered susceptible by the emergence of matching virulence in *Pca* five years or less after release, and similar observations have been made in Canada and in Australia. At least 10 resistance genes derived from *A. sterilis* have been deployed in pure line oat cultivars in Australia, Canada, USA, and elsewhere (viz. *Pc38*, *Pc39*, *Pc48*, *Pc50*, *Pc56*, *Pc58*, *Pc59*, *Pc61*, *Pc62*, *Pc68*; Supplementary Table S1), and in all cases matching virulence was detected very soon following deployment.

The emergence of virulences for genes *Pc50*, *Pc68,* and *Pc91* in Australia provide well documented examples of oat cultivars that were resistant to crown rust at the time of deployment but succumbed soon thereafter. All three genes were deployed only in northern regions of the eastern Australian cereal belt. The dramatic selective force exerted on populations of *Pca* there by cultivars carrying these genes is apparent from the rapid appearance of virulence for each gene, the rapid increase in virulence frequency following gene deployment, and the lack of detectable virulence for each gene in Western Australia where they have not yet been deployed. Gene *Pc68* was first deployed in eastern Australia in 1998 in the forage oat cultivars Graza 68 (syn. AC Assiniboia) and Moola (syn. Dumont 68), both of which likely carry *Pc38* and *Pc39* as well as *Pc68* (Supplementary Table S1). Monitoring for virulence on *Pc68* began in 1993 (Bonnett 1996), and virulence was first detected in 1999 only a year after the gene was first deployed, in a pathotype that combined virulence for *Pc38*, *Pc39* and *Pc68* (Park RF, unpublished). Since then, more than 50 pathotypes virulent for *Pc68* have been detected, all of which are also virulent on *Pc38* and *Pc39*. Monitoring for virulence on *Pc50* began in 1977, and between then and 2007 only a single isolate of one pathotype of *Pca* was detected that was virulent on this gene (Brouwer 1983; Bonnett 1996; Park RF unpublished). Gene *Pc50* was first deployed in Australia in the cultivar Volta, a forage oat released in the east in 2003 (Supplementary Table S1). In 2008, two pathotypes with virulence for *Pc50* were detected, with six additional *Pc50-*virulent pathotypes being detected up to 2020 (Park RF unpublished). Monitoring for virulence on *Pc91* began in 1993 (Bonnett 1996), the gene was first deployed in the forage oat cultivar Drover in 2006, and viruence was first detected in 2010 (Park, 2013). Two years later, two further virulent pathotypes had been detected and approximately 35% of isolates analysed from eastern Australia were virulent for *Pc91*, and by 2020 a further seven pathotypes virulent for *Pc91* had been detected (Park RF unpublished). All pathotypes detected virulent on *Pc91* were also found to be virulent on *Pc38*, *Pc39* and *Pc68*, and one combined virulence on all of these genes with *Pc50*, showing the tremendous evolutionary potential of this asexually reproducing pathogen population. At least 10 other oat cultivars with known or unknown seedling resistance genes to *Pca* were developed and released in Australia from 1991 to 2020 (Amby, Barcoo, Bettong, Cleanleaf, Comet, Culgoa, Gwydir, Nobby, Taipan, Warrego) that were regarded as resistant to crown rust at the time of release but succumbed to the emergence of pathotypes with matching virulence soon after. Multipathotype testing with an array of pathotypes of *Pca* including pathotypes virulent on these cultivars has supported the presence of resistances based on genes *Pc38*, *Pc39*, *Pc48*, *Pc52*, *Pc56*, *Pc59* or *Pc61* singly or in various combinations (Supplementary Table S1). Of interest, genes *Pc38*, *Pc39*, *Pc50*, *Pc52*, *Pc56*, *Pc68* and *Pc91* all appear to have been deployed in various combinations (e.g. cultivars Cleanleaf, Drover, Graza 68, Riel, Volta) and genes *Pc48*, *Pc59, Pc60* and *Pc61* separately in other combinations (e.g. cultivars Amby, Barcoo, Bettong, Nobby). A similar situation was reported for the genes *Pc58*, *Pc59*, *Pc60* and *Pc61* in South America by Leonard and Martinelli (2005), who stated that these genes are most likely to have been introduced into South American oat cultivars from germplasm derived from Texas A&M University and the Coker Seed Company that was distributed in the Quaker Oat International Nursery. In Australia, deploying these two groups of genes separately was paralleled by the emergence of two dominant groups of many pathotypes that are characterised by combinations of virulence for each (Park RF unpublished).

### Multiline cultivars

Multiline cultivars are “re-constitutable composites of phenotypically similar, genetically dissimilar lines” (Jensen 1965). By increasing crop genetic diversity and ensuring that mixture components are relevant to prevailing pathogen populations, such mixtures are believed to buffer against abiotic and biotic stresses (Mundt 2002). Browning and colleagues at Iowa Agriculture and Home Economics Experimental Station and USDA-ARS applied the concept of multiline cultivars to crown rust control in oats by backcrossing different Pc genes into a common genetic background to produce component isolines. Resulting BC_6_ (backcross) fixed lines were mixed to produce a cultivar comprised of between 7 and 12 agronomically similar isolines with different crown rust resistance derived from either *A. sativa* (*Pc5*, *Pc14*) or *A. sterilis* (*Pc36*, *Pc46*, *Pc51*, *Pc52*, *Pc53*, *Pc57*, *Pc58*, *Pc71*; Leonard 2003) (Supplementary Table S1). Fourteen multiline cultivars were developed, registered and released in the 1960s and 1970s under the “E” series (early maturing; Frey et al. 1971a, 1985; Frey and Browning 1976b) and “M” series (midseason maturity; Frey et al. 1971b; Frey and Browning 1976a) plus the multiline cultivar Webster (Frey et al. 1988) (Supplementary Table S1). The multilines were grown on up to 0.4 million hectares in Iowa in the 1970s with no reported losses due to rust (Mundt and Browning 1985). According to Leonard (2003), Multiline E cultivars were still being grown in Iowa on about 15–20% of the acreage sown to oat, Webster continued to be grown in Minnesota into the 1990s, but series “E” cultivars were discontinued due to grower dissatisfaction. Although increases in virulence for genes *Pc36*, *Pc57*, *Pc70* and *Pc71* were seen in some parts of the USA during the 1980s and 1990s, Leonard (2003) speculated that this was not due to the cultivation of multiline cultivars but due to the possible deployment of these genes individually as a result of the use of the isolines used in Webster in breeding programs. This could also account for the use of gene *Pc52* in cultivar Cleanleaf in Australia (Supplementary Table S1).

### The role of wild *Avena* species in the epidemiology of *Pca* and implications for resistance breeding

Genetic diversity in pathogen populations and the size of pathogen populations have both been important determinants of the success in the genetic control of rust pathogens. The eradication of barberry in the USA from 1918 to 1980 was shown to have a large impact on both the oat stem rust pathogen *Pga* and the wheat stem rust pathogen *P. graminis* f. sp. *tritici* (*Pgt*), by delaying epidemic onset, reducing initial inoculum levels, vastly reducing the number of pathotypes, and stabilizing the pathogen population (Roelfs 1982). Long-term studies of *Pgt* in Australia, where sexual recombination is absent, have shown a strong association between the amount of stem rust present and the number of pathotypes detected (Zwer et al. 1992; Park 2007). *Pca* is a heteroecious macrocyclic rust pathogen, which alternates between *Avena* species in the telial stage and *Rhamnus* species (buckthorn) in the aecial stage. While sexual recombination has been documented in the Middle East, Europe and North America (Dinoor 1977; Simons 1985; Wahl 1970), it is absent in Australia where the pathogen reproduces solely via clonal asexual means. Pathogenicity surveys of *Pca* in Australia have been conducted at the University of Sydney since 1936 (Waterhouse, 1952; Baker and Upadhyaya 1955; Luig, 1985; Brouwer and Oates 1980; Park et al. 2000; Park RF, unpublished), and although not continuous, data from the past 70 years have clearly established that Australian populations of this pathogen are pathogenically very diverse despite the absence of sexual recombination (Luig 1985). Over the 40 year period 1979 to 2019, 107 pathotypes of *Pca* were detected compared to 23 of the oat stem rust pathogen *P. graminis* f. sp. *avenae* (Pga), 25 of the wheat stem rust pathogen *P. graminis* f. sp. *tritici*, 25 of the wheat leaf rust pathogen *P. triticina*, 24 of the wheat stripe rust pathogen *Pst*, and 31 of the barley leaf rust pathogen *P. hordei* (Park, RF unpublished).

The high degree of pathogenic diversity in *Pca* in Australia in the absence of sexual recombination is most likely a function of the large pathogen populations maintained on extensive wild oat communities. In addition to *A. sativa*, *Pca* also infects ubiquitous wild oats (*A. barbata*, found mainly along roadsides and waste areas; *A. fatua*, which predominates in southern Australia; *A. sterilis* subsp. *ludoviciana*, which is more prevalent in northern New South Wales (NSW) and Queensland (Qld)). Studies have shown that the composition of isolates of *Pca* originating from cultivated and wild oats in Qld and NSW do not differ significantly (Oates et al. 1983; Park et al. 2000), showing the importance of both host systems in the epidemiology and population dynamics of *Pca* there. For example, the cultivar Cleanleaf (*Pc38*, *Pc39*, *Pc52*; Supplementary Table S1) was released in this region in 1992. Virulence on Cleanleaf was first detected in two pathotypes in 1995, and by 1999 a further four had been detected (Park et al. 2000). Following initial detection, virulence for Cleanleaf increased rapidly in Qld and northern NSW, with at least 40% of the isolates identified from 1996 to 1998 being virulent on this cultivar (Park et al. 2000). Furthermore, from 1996 to 1999 the frequency of isolates virulent on Cleanleaf among isolates of *Pca* obtained from cultivated oats and wild oats were very similar, clearly illustrating the close relationship between populations of *Pca* on these two host groups (Park et al. 2000).

Detailed tests of Australian pathotypes of *Pca* have shown the presence of virulence for most designated ASR genes (Tables 1 and 2). Apart from stocks carrying 17 ASR genes that have not been tested, virulence has been detected for all others except Glabrota (*Pc18*, *Pc29*) and CI 3815 (*Pc19*, *Pc30*, *Pc81*-*85*). Many of the designated *Pc* genes for which virulence has been detected have not been deployed in agriculture, not only in existing pathotypes but also in newly detected presumed mutational derivatives of existing pathotypes. In some cases, virulence for these genes has been detected rarely (e.g. *Pc63*, *Pc94*), whereas in other cases it has become very common after being first detected (e.g. *Pc36*, *Pc46*, *Pc92*). The presence of these virulences could be due to random mutation followed by hitch-hiking, the increased frequency of certain virulence combinations due to association with other advantageous triats under direct selection, during asexual reproduction (Brown et al. 1997), or due to the presence of the genes in wild oat populations. Burdon et al. (1983) demonstrated the common occurrence of uncharacterised resistance to four pathotypes of *Pca* in 21 populations of wild tetraploid and hexaploid oats collected from 13 sites across NSW, with populations from the more rust prone northern NSW being significantly more resitant compared to those from the south. Such resistance in wild oat communities would be expected to exert selection pressure on populations of *Pca* in much the same way as is exerted by cultivated hexaploid oat.

### Challenges, future directions, and prospects

#### The problem of pathogenic diversity in *Pca*

In many regions where race analysis is undertaken for cereal rust pathogens, *Pca* is the most pathogenically variable. Even in places such as Australia where sexual recombination is absent, far more pathotypes of *Pca* are detected than for any other cereal rust pathogen. The detection of virulence for *Pc68* in Australia in 1999, only a year after it was first deployed, and of more than 50 *Pc68*-virulent pathotypes over the ensuing 20 years, is a clear example of the variation this pathogen can generate in the absence of sexual recombination. In comparison, virulence in Australia in the similarly asexually reproducing wheat stem rust pathogen *Pgt* for gene *Sr38* was first detected in 2001; although this was just three years after *Sr38* was first deployed in Australia, only five further pathotypes with virulence on this gene had been detected up to 2020 (Park RF, unpublished) despite similar numbers of differentials being used to identify pathotypes in both pathogens (30 for *Pca*, 29 for *Pgt*). The extent of variation in *Pca* in the absence of sexual recombination is staggering, and must be principally due to the large pathogen populations harboured by naturalised weedy wild oats.

The removal of more than 99 million barberry bushes in the USA between 1917 and 1967 and its effect on stem rust on both wheat and oat demonstrate that it is possible to undertake large-scale eradication of noxious weeds to improve disease control. In regions where buckthorn plays a role in the life cycle of *Pca*, large-scale eradication could be undertaken, but as in the case of the barberry eradication campaign, would require significant resourcing and ongoing efforts to ensure awareness of the danger posed to cereal production to prevent reestablishment (Leonard 2001). Even if alternate hosts such as buckthorn are eradicated in oat growing regions, it is hard to see how extensive natural and naturalised wild oat populations that occur in many oat growing regions could be removed or reduced to an extent that would have a significant impact on *Pca*. In the Australian wheat: *Pgt* system, there is evidence that the widespread cultivation of genetically resistant wheat cultivars reduced pathogen population size and diversity, stabilising resistance and the pathogen population (Zwer et al. 1992). Given that few non-cereal grass species are susceptible to *Pgt* in Australia, resistant wheat cultivars not only control rust in crops, but also reduce opportunities for oversummering (the “green bridge”) and early season buildup (the “green ramp”). In contrast, the widespread occurrence of wild oats as ancillary hosts means that any strategy to control *Pca* must be based on the assumption of a continuing, large and variable prevailing pathogen population with high evolutionary potential.

### Monitoring pathogen virulence and the promise of sequence-base pathogenomics

Race (pathotype) surveys of cereal rust pathogens have been conducted in many parts of the world since the early 1900s. The only way to identify rust pathotypes remains greenhouse virulence testing using genotypes (“differentials”) with different resistance genes. In view of the very high level of pathogenic variability in *Pca*, and the lack of durability of ASR genes, the question could be asked as to whether pathotype (race) analysis should be undertaken for this pathogen at all. Identifying what pathotypes are present where and in what frequencies by either greenhouse testing, or in future possibly genotyping, while important, is only part of the role pathotype surveys play in rust control. When conducted in a robust and relevant way, surveys underpin all gene-based rust control strategies, from devising breeding strategies, to gene discovery, to post-release stewardship of resistance resources (Park 2015).

Rust response phenotypes are relatively easy to record and high throughput phenotyping systems have been in place in rust laboratories around the world for many years. To be relevant to agriculture, such phenotyping systems are entirely reliant on pathotype surveys to provide the most important, fully characterised isolates (defined pathotypes) for use in identifying new sources of resistance and screening breeding material. The use of minor gene APR in breeding wheat for resistance to all three rust pathogens has received increased attention over the past 50 years (e.g. Singh et al. 2011). The most efficient way to separate the effects of ASR from APR has been to use pathotypes that render any ASR genes present ineffective by matching virulence. This approach has been used extensively by staff and students at the Plant Breeding Institute University of Sydney in screening tens of thousands of lines of oats to identify those carrying useful levels of APR to crown rust. Being able to monitor and know what ASR genes are present in breeding programs is also important to prevent the inadvertent selection of ASR genes that are known to be non-durable. These include “fossil” resistance genes, genes present in germplasm that are occasionally revealed by the recurrence of previously rare avirulence or occurrence of unusual avirulences. For example, a pathotype of *P. triticina* first identified in Australia in 2004 was found to be avirulent on an ASR gene, *Lr73*, present in the rust susceptible genotype Morocco and nine Australian wheat cultivars (Park et al. 2014). Being able to recognise *Lr73* has been important in preventing its unintentional selection in resistance breeding. Knowing what pathotypes are present and in what frequency will also be critical if multilines are used in future (see below).

It is impractical to include all currently designated/ temorarily designated alleles conferring resistance to *Pca* in a differential set that is used routinely. It is also of little value as many of the genes are defeated or have not been used in agriculture. As discussed in detail by McIntosh et al. (1995), the reasons for including or excluding differential genotypes in a defined differential set are varied, and factors such as geographical differences in virulence frequencies and the resistance genes deployed in agriculture limit international agreement on what genotypes should be used. Chong et al. (2000) proposed a set of 16 differential genotypes as the basis of a North American system for naming pathotypes of *Pca*. Most if not all of these are also used as differentials in Australia (Park RF unpublished), the Czech Republic (Klenová-Jiráková et al. 2010), Poland (Paczos-Grzeda and Sowa 2019), South Africa (Boshoff et al. 2020) and Spain (Sánchez-Martín et al. 2012), and hence can be considered as core international pathogenicity testers for *Pca*. Excluding *Pc54* and *Pc64*, the differential gene in 14 of these genotypes has been deployed in agriculture in at least one cultivar (Supplementary Table S1). Genes that are not represented, but which have been deployed, include *Pc1*, *Pc3*+*Pc4*, *Pc10*, *Pc13*, *Pc14*, *Pc61*, *Pc91*, *Pc94*, plus genes in older sources of resistance (Anthony/Bond//Boone, Bond, Garry, Landhafer, Santa Fe, Trispernia, Ukraine, Victoria). Many of these are either present in currently grown oat cultivars or still provide useful discrimination of local populations of *Pca*, and are thus used by some groups as regional supplemental differentials. Should APR genes be deployed in commercial oat cultivars, it may be necessary to monitor virulence on these as not all APR genes are durable (Park and McIntosh 1994).

### Designated *Pc* loci

Based on the resistance loci catalogued to date, it would appear that the diversity of resistance to *Pca* in *A. sativa* is low, and to *Pga* it appears to be even lower with only 12 loci being designated from genotypes of *A. sativa* (Gnanesh et al. 2014). The 92 loci that confer resistance to *Pca* in *Avena* that have been designated so far may seem to be a large number, however to date 78 distinct loci conferring resistance to *P. triticina* have so far been mapped robustly and designated in wheat.

Allelic relationships between many of the designated *Pc* genes are unknown, and it is likely that at least some are duplicates, or even triplicates. Although 19 loci have been designated from the diploid *A. strigosa*, only three or possibly four are available in stable hexaploid lines and there is convincing evidence that some of these represent the same gene, raising the question as to how diverse resistance to *Pca* in *A. strigosa* actually is. The recent release of chromosome scale assemblies for the two diploid species *A. atlantica* and *A. eriantha* (Maughan et al. 2019) and the hexaploid *A. sativa* genotype OT3098 (https://wheat.pw.usda.gov/GG3/graingenes_downloads/oat-ot3098-pepsico) have provided invaluable genetic resources that will unquestionably accelerate efforts to map all designated *Pc* genes for which unambigiuous genetic stocks exist, and to assess potential allelism between them. It is to be expected that this will lead to changes in the numbering of at least some designated loci, and possible deletion of others from the catalogue, as has occurred in wheat (e.g. the deletion of *Sr1* due to synonymy with *Sr9d*, and of *Sr3* and *Sr4* due to a lack of single gene stocks; McIntosh et al. 1995).

### Searches for new sources of resistance

Simons (1985) stated that “*It is now apparent that there is little point in studying the genetics of resistance to* P. coronata *unless the resistance has some practical usefulness*.” Most efforts to find new sources of resistance to *Pca* until now have focused on ASR genes, and most of these genes have come from species of *Avena* other than *A. sativa*. Searches for new sources of resistance in alien species will no doubt uncover new ASR genes. Cabral and Park (2014) examined resistance to *Pca* in 97 accessions of *A. strigosa* from North America, South America and Europe, finding that 20 lacked detectable resistance to the pathotypes used, 18 carried the Saia resistance, one *Pc94*, and only three that were resistant to all pathotypes used. A second study using SSRs showed that the *A. strigosa* accessions were 86% genetically similar, so this study may not be a true reflection of the genetic variability available in this species (Cabral et al. 2013). The accessions proved to be very difficult to work with and very few introgression stocks could be produced (Aung et al. 2010; Cabral et al. 2011; Cabral and Park 2014, 2016). Any attempt to introgress rust resistance from diploid or tetraploid *Avena* species to hexaploid oat, in the absence of transgenic approaches, will take at least 10 years to be of benefit to oat growers. It should also be noted that any resistance genes sourced from *Avena* species that are weeds could well be already present in weedy wild oat communities in regions where the resistance is deployed in agriculture.

Sánchez-Martín et al. (2012) screened 159 *Avena* accessions including *A. sativa* (subspp. *sativa* and *strigosea*) landraces and cultivars. Eight landraces and four cultivars showed an elevated degree of resistance according to macroscopic components at seedling stage under controlled conditions and further histological analysis showed a set of different resistance responses acting at susccesive stages of the infection process. Later, field experiments of the commercial varieties at six contrasting locations along Mediterranean Basin, including Spain, Tunisia, Egypt and Palestinian Territories, showed that some of these varieties were consistently resistant across these different environments (Sánchez-Martín et al. 2014). Interestingly, these accessions were characterised by a high level of pre-penetration resistance and/ or early aborted colonies not associated with host cell necrosis. This study highlights how knowledge of resistance mechanisms/responses may enable the use of plant genotypes able to stop the pathogen at different developmental stages reducing the pressure on ASR genes (usually related to hypersensitive response) and may prove more durable and difficult to overcome by evolving pathogenic races (Rubiales and Niks 2000).

Six designated loci (*Pc27*, *Pc28*, *Pc69*, *Pc72*, *Pc73*, *Pc74*) are reported as conferring APR to crown rust. Several other oat cultivars (Ajax, Beaver, Erban, Lodi, Portage, Red Rustproof, Rodney, Santa Fe, Ukraine) and accessions (PI 174545, PI 18783, PI 197278, PI 174544, PI 179279, MN841801, MN841804) have been reported to carry uncharacterised APR to crown rust. The latter are especially promising as potential sources of effective and durable resistance given that they displayed stable resistance in a buckthorn nursery over seven years (Leonard 2002), and one or both lines have shown good levels of APR under field conditions in Canada, the USA and Australia in subsequent studies. Both lines have also been shown to induce premature telial formation, truncating urediniospore production, reducing inoculum load, and hence functioning as a form of resistance (viz. MN841801 and MN841804). These lines were distributed with the 2000 Quaker International Oat Nursery and were shown to induce premature telial formation under Australian field conditions (Roake J and Park RF unpublished). This type of resistance may hold some promise in future resistance breeding efforts, especially as a component of polygenic minor APR.

In addition to these sources of APR to *Pca*, integrated greenhouse seedling tests and field adult plant tests of more than 100,000 oat genotypes at the University of Sydney’s Plant Breeding Institute over the past 25 years have identified 385 lines of *A. sativa* with reasonable agronomic performance that carry levels of APR to *Pca* varying from low to very high (Haque 2004; Issa 2005; Park RF, Singh D, and Roake J, unpublished). Combined, these studies demonstrate that APR to crown rust in hexaploid oat is not uncommon, and although the genetic diversity of the resistance found to date is not yet well understood, it is known that multiple genes of additive effects are involved in some cases and that some of these have been stable over time and in different environments, making them potentially valuable in breeding for resistance to *Pca*.

Studies under controlled conditions have shown that APR genes are switched on at different growth stages, some soon after seedling growth stages and others not until flag leaf emergence (Park and McIntosh 1994). Just how and at what growth stages resistance genes are expressed are important considerations in ensuring adequate protection from diseases, especially so if APR is used in grazing oat, in which the early onset of resistance will be essential because of the implications of withholding periods for fungicides in grazing.

#### Resistance gene deployment

The concept of combining resistance genes to bestow greater durability was proposed by researchers many years ago (e.g. Watson and Singh 1952). The development of markers linked to resistance genes continues to improve the efficiency by which multiple gene resistances can be assembled (e.g. *Pc68*, Chen et al. (2006), Satheeskumar et al. (2011); *Pc71* (Bush and Wise 1998); *Pc92* (Rooney et al. 1994); *Pc94* (Chong et al. 2004) and *Pc91* (McCartney et al. 2011)). The problem of pathogen variability, however remains; if this approach is applied to pyramiding genes conferring ASR to *Pca*, it will be of critical importance to ensure that none are deployed singly or occur in a natural state in wild oat populations. The use of single genes provides the “stepping stones” needed for the pathogen to acquire the virulences needed to render multigenic resistances ineffective. This was documented in Australia when cultivars carrying *Pc39* (Quamby, released 1990), *Pc52* (Cleanleaf, 1992), *Pc38* and *Pc39* (Riel, 1993), *Pc68* (Graza 68, 1998), *Pc50* (Volta, 2003), and *Pc91* (Drover, 2006) were released and *Pca* acquired sequential matching virulence resulting in a single pathotype combining virulence for all six ASR genes in about 25 years (Park RF unpublished). Although no rust resistance gene has been isolated from oat to date, the availability of reference genomes and the availability of non-map-based approaches to gene cloning (e.g. Steuernagel et al. 2017) will no doubt fast-track efforts to do so. The recent availability of reference genomes for hexaploid and diploid oat will undoubtedly also accelerate efforts to discover, characterise and develop high throughput diagnostic markers to introgress resistance to *Pca* into high yielding adapted oat germplasm. The common occurrence of minor gene, additive APR to *Pca* in hexaploid oat germplasm raises the possibility of pyramiding several such genes to give high levels of resistance, an effort that will be greatly expedited by the development of high throughput diagnostic markers based on gene sequences.

Luo et al. (2021) recently published a protocol to stack cloned ASR and APR genes to *Pgt* in a single cassette and transform them into wheat as a single locus. The authors stressed the importance of strategic deployment of such stacks to retain their effectiveness, highlighted by the detection of a pathotype of *Pgt* combining virulence for three of the four stacked ASR genes in Europe despite none having been deployed in agriculture.

An important consideration in combining resistance genes is resistance gene suppression. Hurni et al. (2014) demonstrated that the susceptibility allele *Pm3C*, and three resistance alleles (*Pm3a*, *Pm3b* and *Pm3f*) all suppressed the rye derived resistance gene *Pm8,* which had been introgressed into wheat. The strength of the suppression was shown to vary with the *Pm3* allele used, raising the possibility that gene editing could be used to modify alleles so that suppression did not occur. Wilson and McMullen (1997) showed that *Pc38* suppressed *Pc62*, and Chong and Aung (1996) found that *Pc38* suppressed *Pc94*. While these examples may be the exception, it remains an important consideration in control strategies based on combining resistance genes.

An alternative to multi-gene cassettes in a single cultivar is to use transformation to introduce different genes or gene combinations into a single background, and mix to produce multiline cultivars. The oat multilines with resistance to crown rust developed by the Iowa group were based on common backgrounds that were developed well before the multiline was release. For example, multilines E76 and E77 were based on the recurrent parental line CI 8044, developed from the cross Clintland/Garry 5, with the original cross being made in 1954 and eventual release of the multiline cutivars in 1976 and 1977 (Frey et al. 1985). Shortening the 20 year period between making the first cross and cultivar release by using transformation technologies would ensure future multiline cultivars compete with genetic gains made in pure line cultivar breeding programs, and ensure quality traits matched to market demands. An efficient transformation system and supply of cloned resistance genes in oat may make this possible. In the absence of acceptance of transgenic oat, accelerated generation breeding approaches (Gonzalez-Barrios et al. 2020) combined with marker assisted selection and genomic selection/ prediction (Asoro et al. 2011; Rio et al. 2021) could be used.

Mundt (2002) stressed the importance of functional diversity in multiline cultivars; randomly chosen mixtures will not necessarily provide adequate disease control, and in order to be effective mixture components need to be relevant, or functional, against a pathogen population. Of the nine genes used to develop the oat multilines at Iowa, combined virulence exists for six in a single pathotype in Australia despite none of these genes being deployed there (Park RF unpublished). Understanding the virulence compositon of populations within an epidemiological region is therefore critical in ensuring that the components and their frequencies within mixtures bestow the best protection possible against prevailing pathotypes. Integrating pathogen surveillance with gene deployment in this way would be analogous to the approach used to mass vaccinate chickens against prevailing variants of the infectious bronchitis virus (Jordan 2017).

There is evidence that genetic “background” can influence durability (*eg*. The *pvr2*^*3*^ gene in pepper, Palloix et al. 2009; *Lr24* in wheat in Australia, Park et al. 2000), supporting the strategy of using reputedly durable sources of rust resistance as “background” resistance, to which other resistance genes are added (McIntosh 1988). The discovery of three durable pleiotropic minor gene (APR) loci in wheat that provide resistance to three rust pathogens and powdery mildew, and the development of diagnostic gene-based markers (*Lr67*/*Yr46*/*Sr55*; *Lr34*/*Yr18*/*Sr57*) or closely linked markers (*Lr46*/*Yr29*/*Sr58*) has been a huge advance in efforts to breed for durable rust resistance in this crop. These markers have allowed for the selection of these minor APR genes in breeding populations and their use as background or backbone resistance on top of which ASR genes have been pyramided. These markers have also made discovering other sources of minor APR faster by allowing genotypes carrying APR to be genotyped and attention focused on lines lacking these genes but carrying useful levels of APR. Whether or not such pleiotropic genes exist in oat is unknown, but given their importance in wheat efforts to discover new sources of resistance in oat should take this possibility into consideration.

### Enduring rust control

As stated by Browing and Frey (1969), it is populations of plants that feed increasing populations of people, not individual plants. While the development and use of oat cultivars with reputedly durable sources of resistance to crown rust will be foundational in attempts to achieve enduring crown rust control, it must be noted that not all durable resistance protects against yield loss (e.g. Ma and Singh 1996), and that durable resistance may not remain so forever (Johnson 1984). Ensuring that the durable resistance sources used to develop crown rust resistant oat cultivars are genetically diverse and bestow levels of resistance that prevent yield loss and slow epidemic development will be vital in reducing the impact of this disease in oat production, and in particular buffering against potential future changes in *Pca*.

The use of diverse sources of resistance has been a critical aspect of enduring stem rust control in wheat in Australia for the past 50 years (Park 2007). Stem rust resistance breeding has been underpinned by close monitoring of the pathogen, and integrated with practices including avoiding the cultivation of highly susceptible cultivars, not overlapping crops and optimising the break between cropping cycles, green bridge destruction, and at times the use of fungicides. Achieving enduring control of oat crown rust will require a similar integrated whole of system approach based on a foundation of durable resistance. This could include manipulating sowing date and crop phenology, which have been shown to reduce crown rust severity. Simons and Michel (1968) found that early maturing oat varieties tend to be less damaged than do later maturing varieties, probably because they ripen before the disease becomes fully established. Fleischmann and McKenzie (1965) reported less crown rust damage in the oat cultivar Garry, which carries the APR genes *Pc27* and *Pc28*, when it was planted earlier than usual. The durability of any resistance to crown rust deployed in agriculture will continue to be challenged by the ongoing presence of large and variable populations of *Pca* in wild oat communities in many of the world’s oat growing regions and of a functional sexual stage in some regions. The oat: *Pca* system may therefore also provide important opportunities to understand how best to integrate durable resistance with non-gene-based control strategies to protect important genes and achieve enduring crop disease control.

## Supplementary Information

Below is the link to the electronic supplementary material.

Supplementary file1 (XLSX 42 KB)

Supplementary file1 (DOCX 64 KB)
