# Supplementary material for: Breeding oat for resistance to the crown rust pathogen Puccinia coronata f. sp. avenae: achievements and prospects
Source: Theor Appl Genet. 2022 Jun 4;135(11):3709–34. doi: 10.1007/s00122-022-04121-z (PMC9729147; doi:10.1007/s00122-022-04121-z)
Supplement: Supplementary file 2 — Supplementary file1 (DOCX 64 KB) [file 122_2022_4121_MOESM2_ESM.docx]

**Table 3**. Markers associated with designated (catalogued) genes conferring resistance to *Puccinia coronta* f. sp. *avene* in oat. GrainGenes was used to identify sequence accession of probe and top 3 BLAST hits (E value < 10-5) physical positions in the chromosome assembly PepsiCo OT3098 Hexaploid Oat v2 pseudomolecules (2021)

| Gene associated | | Original study | | | | | | | | PepsiCo OT3098 v2 BLAST | | | | | | | | |  |
| --- | --- | --- | --- | --- | --- | --- | --- | --- | --- | --- | --- | --- | --- | --- | --- | --- | --- | --- | --- |
|  |  | Carrier | Reference | Population ID^1^ | Population Type | Probe | Genetic distance from Pc gene | Marker type | Comparitive mapping (Refrence study)^2^ | Sequence Accession | No. hits | Query coverage (%) | Total Score | E value | Identity (%) | Chromosome | Start (Mbp) | Stop (Mbp) |  |
| *Pc38* | | Pendek38 | Wight et al. (2004) | Pendek4838 | F2:3 RILs | cdo673b | 2 cM | RFLP | KO_17 (6.46 cM)^d^ | BE439193 | 3 | 84 | 422 | 2.82x10^-103^ | 99 | 2A | 411.01 | 411.01 |  |
|  |  |  |  |  |  |  |  |  |  |  |  | 84 | 412 | 1.46x10^-100^ | 98 | 7D | 403.77 | 403.77 |  |
|  |  |  |  |  |  |  |  |  |  |  |  | 84 | 372 | 1.05x10^-89^ | 94 | 4C | 131.98 | 131.98 |  |
|  |  |  |  |  |  | cdo341 | 2 cM | RFLP | KO_17 (5.94cM)^d^ | BE439188 | 3 | 96 | 676 | 4.61x10^-172^ | 100 | 2D | 29.54 | 29.54 |  |
|  |  |  |  |  |  |  |  |  |  |  |  | 96 | 568 | 9.52x10^-143^ | 94 | 7D | 28.77 | 28.77 |  |
|  |  |  |  |  |  |  |  |  |  |  |  | 96 | 462 | 5.63x10^-114^ | 83 | 7A | 112.63 | 112.63 |  |
|  |  |  |  |  |  | cdo1199b | 2 cM | RFLP | KO_17 (15.2 cM)^d^ | BE439152 | 3 | 100 | 546 | 6.68x10^-137^ | 100 | 4C | 219.85 | 219.85 |  |
|  |  |  |  |  |  |  |  |  |  |  |  | 100 | 469 | 9.93x10^-116^ | 94 | 7D | 341.08 | 341.08 |  |
|  |  |  |  |  |  |  |  |  |  |  |  | 100 | 469 | 9.93x10^-116^ | 94 | 7A | 439.37 | 439.37 |  |
|  |  |  |  |  |  | cdo608b | 2 cM | RFLP | KO_17 (5.28 cM)^d^ | CN180778 | 19 | 100 | 2164 | 0 | 98 | 5D | 18.24 | 18.24 |  |
|  |  |  |  |  |  |  |  |  |  |  |  | 100 | 1752 | 0 | 98 | 5A | 49.03 | 49.03 |  |
|  |  |  |  |  |  |  |  |  |  |  |  | 98 | 1619 | 0 | 94 | 4C | 604.73 | 604.73 |  |
|  |  |  |  |  |  | cdo1467a | 2 cM | RFLP | KO_17 (6.91 cM)^d^ | BE439161 | 3 | 100 | 580 | 4.92x10^-146^ | 93 | 7A | 84.91 | 84.91 |  |
|  |  |  |  |  |  |  |  |  |  |  |  | 100 | 526 | 2.23x10^-131^ | 91 | 7D | 4.08 | 4.08 |  |
|  |  |  |  |  |  |  |  |  |  |  |  | 100 | 506 | 6.00x10^-126^ | 88 | 5C | 576.01 | 576.01 |  |
|  |  |  |  |  |  | isu1146bdi | 2 cM | RFLP | KO_17 (6.91 cM)^d^ | CN180780 | 3 | 100 | 1488 | 0 | 99 | 7D | 4.07 | 4.08 |  |
|  |  |  |  |  |  |  |  |  |  |  |  | 100 | 1365 | 0 | 94 | 7A | 84.91 | 84.91 |  |
|  |  |  |  |  |  |  |  |  |  |  |  | 100 | 1286 | 0 | 92 | 5C | 576.01 | 576.01 |  |
|  |  |  |  |  |  | cdo1340 | 2 cM | RFLP | KO_17 (10.7 cM)^d^ | CN180769 | 3 | 100 | 1632 | 0 | 99 | 7D | 183.51 | 183.51 |  |
|  |  |  |  |  |  |  |  |  |  |  |  | 100 | 1622 | 0 | 98 | 7A | 292.98 | 292.98 |  |
|  |  |  |  |  |  |  |  |  |  |  |  | 94 | 969 | 0 | 83 | 7C | 273.84 | 273.84 |  |
|  |  |  |  |  |  | wg420a | 2 cM | RFLP | KO_17 (6.62 cM)^d^ | CN180784 | 9 | 94 | 9645 | 0 | 80 | 7D | 433.43 | 520.33 |  |
|  |  |  |  |  |  |  |  |  |  |  |  | 94 | 2162 | 0 | 76 | 6C | 2.66 | 39.59 |  |
|  |  |  |  |  |  |  |  |  |  |  |  | 93 | 571 | 1.35x10^-120^ | 70 | 4D | 25.09 | 443.20 |  |
|  |  |  |  |  |  | CDO113 | 4 cM | SCAR | KO_22_44+18 (68 cM)^d^ | BE439054 | 3 | 100 | 310 | 1.04x10^-39^ | 90 | 4D | 355.03 | 355.03 |  |
|  |  |  |  |  |  |  |  |  |  |  |  | 99 | 296 | 3.64x10^-39^ | 88 | 4A | 398.87 | 398.87 |  |
|  |  |  |  |  |  |  |  |  |  |  |  | 94 | 150 | 6.17x10^-30^ | 91 | 3C | 617.78 | 617.78 |  |
|  |  | Dumont |  | OT328Du |  | cdo1385c | 7cM | RFLP | KO_7_10_28 (78.9cM)^d^ | BE439299 | 11 | 94 | 1868 | 2.29x10^-176^ | 95 | Un | 42.67 | 52.36 |  |
|  |  |  |  |  |  |  |  |  |  |  |  | 91 | 506 | 7.02x10^-126^ | 89 | 1C | 89.02 | 89.02 |  |
|  |  |  |  |  |  |  |  |  |  |  |  | 50 | 278 | 5.41x10^-64^ | 90 | 4C | 526.02 | 526.02 |  |
| *Pc39* | | AC Assiniboia, AC Medallion | Zhao et al. (2020a) | AsbMN, MedMN | F8:9 RILs, F6:7 RILs | avgbs_126086.1.41 | 1.7 cM, 0.4 cM | SNP | Mrg11 (1D) (3.7 cM)^h^ | avgbs_126086.1.41 | 2 | 100 | 108 | 7.32x10^-19^ | 93 | 4C | 5.05 | 5.05 |  |
|  |  |  |  |  |  |  |  |  |  |  |  | 100 | 108 | 7.32x10^-19^ | 93 | 6A | 428.89 | 428.89 |  |
|  |  |  |  |  |  | GMI_ES15_c276_702 | 0.3 cM, 0.4 cM |  |  | GMI_ES15_c276_702 | 2 | 100 | 234 | 1.21x10^-52^ | 98 | 6A | 435.94 | 435.94 |  |
|  |  |  |  |  |  |  |  |  |  |  |  | 100 | 193 | 3.03x10^-41^ | 91 | 5D | 486.95 | 486.95 |  |
|  |  |  |  |  |  | GMI_ES01_c12570_390 | co-segregating |  |  | GMI_ES01_c12570_390 | 3 | 83 | 199 | 7.13x10^-43^ | 99 | 4C | 1.95 | 1.95 |  |
|  |  |  |  |  |  |  |  |  |  |  |  | 83 | 199 | 7.13x10^-43^ | 99 | 6A | 433.39 | 433.39 |  |
|  |  |  |  |  |  |  |  |  |  |  |  | 83 | 137 | 4.81x10^-26^ | 87 | 5D | 484.61 | 484.61 |  |
|  |  | Celer | Sowa and Paczos-Grzeda (2020) | CelerSTH9210 | F2 and F2:3 RILs | oPt-17172 | 5.3cM | DArT | Mrg11 (3.7-6.7 cM)^h^ | oPt-17172 | 4 | 100 | 1044 | 0 | 100 | 4C | 2.26 | 2.26 |  |
|  |  |  |  |  |  |  |  |  |  |  |  | 68 | 587 | 4.16x10^-124^ | 83 | 6A | 432.91 | 433.10 |  |
|  |  |  |  |  |  |  |  |  |  |  |  | 98 | 785 | 3.00x10^-94^ | 76 | 5D | 484.20 | 484.20 |  |
|  |  |  |  |  |  | SCAR_RAPD_H11 | 2.2 cM | SCAR |  | SCAR_RAPD_H11 | 1 | 87 | 1512 | 7.52x10^-144^ | 82 | 5D | 494.95 | 495.00 |  |
|  |  |  |  |  |  | 3456272 | 10.3 cM | DArT |  | 3456272 | 1 | 62 | 135 | 5.89x10^-8^ | 91 | 6A | 440.15 | 440.21 |  |
|  |  |  |  |  |  | 3454401 | 12 cM |  |  | 3454401 | 1 | 99 | 64 | 7.18x10^-7^ | 79 | 6A | 440.15 | 440.15 |  |
|  |  |  |  |  |  | 3456624 | 0.37 cM |  |  | 3456624 | 1 | 100 | 84 | 2.7x10^-12^ | 81 | 6A | 434.06 | 434.06 |  |
|  |  | - | McNish et al. 2020 | UMOF | GWAS panel | avgbs_120698.1.15 | - | SNP | Mrg05 (10cM)^h^ | avgbs_120698.1 | 1 | 100 | 125 | 3.32×10^-23^ | 98 | 6A | 4.30 | 4.30 |  |
| *Pc39, Pc71* | | Pendek39 | Wight et al. (2004), Bush and Wise (1998) | Pendek3948, D526BC | F2:3 RILs, BC1F2 | umn5032 | co-segregating, 2.6 cM | RFLP | KO_37^d^, LG 11^b^ | CL524684 | 2 | 64 | 9302 | 0 | 92 | 6A | 440.09 | 440.09 |  |
|  |  |  |  |  |  |  |  |  |  |  |  | 5 | 104 | 3.82x10^-16^ | 88 | 2D | 479.15 | 479.15 |  |
|  |  | Pendek39 and OT328Du, C237-89 | Wight et al. 2004, Bush and Wise (1998) | Pendek3948 and OT328Du, D526BC |  | ISU2287 | co-segregating and 6 cM, 3.1 cM | RFLP | KO_37^d^ , LG 11^b^ | CN180782 | 12 | 100 | 1073 | 0 | 99 | 4A | 409.05 | 409.05 |  |
|  |  |  |  |  |  |  |  |  |  |  |  | 100 | 1038 | 0 | 97 | 4D | 364.03 | 364.03 |  |
|  |  |  |  |  |  |  |  |  |  |  |  | 79 | 721 | 0 | 93 | 3C | 632.50 | 632.50 |  |
| *Pc71* | | C237-89 | Bush and Wise (1998) | D526BC | BC1F2 | CDO783 | 0.2 cM | RFLP | LG 11^b^ | BE439158 | 3 | 100 | 819 | 6.92x10^-157^ | 90 | 4C | 6.20 | 6.21 |  |
|  |  |  |  |  |  |  |  |  |  |  |  | 100 | 820 | 6.92x10^-157^ | 86 | 6A | 427.08 | 427.89 |  |
|  |  |  |  |  |  |  |  |  |  |  |  | 100 | 787 | 1.86x10^-113^ | 83 | 5D | 477.13 | 477.15 |  |
|  |  |  |  |  |  | BCD1270 | 0.4 cM | RFLP | LG 11^b^ | BE438937 | 3 | 74 | 203 | 4.46×10-^24^ | 84 | 6A | 420.03 | 420.03 |  |
|  |  |  |  |  |  |  |  |  |  |  |  | 75 | 205 | 1.56×10-^23^ | 84 | 5D | 467.25 | 467.26 |  |
|  |  |  |  |  |  |  |  |  |  |  |  | 74 | 198 | 1.90×10-^22^ | 83 | 4C | 15.77 | 15.77 |  |
|  |  |  |  |  |  | CDO665 | 1.1 cM | RFLP | LG 11^b^ | BE439169 | 3 | 100 | 620 | 5.64×10^-126^ | 99 | 5D | 459.51 | 459.51 |  |
|  |  |  |  |  |  |  |  |  |  |  |  | 99 | 554 | 2.10×10^-112^ | 95 | 6A | 414.82 | 414.82 |  |
|  |  |  |  |  |  |  |  |  |  |  |  | 99 | 539 | 4.63×10^-108^ | 94 | 4C | 21.40 | 21.40 |  |
|  |  | - | Klos et al. (2017) | CORE | GWAS panel | GMI_DS_LB_270 | - | SNP | Mrg05 (135.5 cM)^g^ | GMI_DS_LB_270 | 3 | 100 | 239 | 9.91×10^-54^ | 99 | 4C | 6.08 | 6.08 |  |
|  |  |  |  |  |  |  |  |  |  |  |  | 100 | 239 | 9.91×10^-54^ | 99 | 6A | 427.99 | 427.99 |  |
|  |  |  |  |  |  |  |  |  |  |  |  | 100 | 239 | 1.38×10^-45^ | 94 | 5D | 477.43 | 477.43 |  |
| *Pc53* | *PcKM* | 6-112-1-15 | Admassu-Yimer at al. (2018a) | Pc53Pc50 | F5:6 RILs | GMI_ES15_c12065 | 9 cM | SNP | Mrg08 (70.4 cM)^g^ | GMI_ES15_c12065_114 | 3 | 100 | 239 | 9.91×10^-54^ | 99 | 2D | 195.67 | 195.67 |  |
|  |  |  |  |  |  |  |  |  |  |  |  | 100 | 218 | 2.66x10^-48^ | 95 | 2A | 404.90 | 404.90 |  |
|  |  |  |  |  |  |  |  |  |  |  |  | 100 | 192 | 3.03×10^-41^ | 90 | 2C | 560.38 | 560.38 |  |
|  |  |  |  |  |  | GMI_ES17_c19330 | 9 cM | SNP | Mrg08 (70.2 cM)^g^ | GMI_ES17_c19330_272 | 6 | 79 | 184 | 4.50x10^-39^ | 97 | 3D | 355.07 | 355.07 |  |
|  |  |  |  |  |  |  |  |  |  |  |  | 79 | 294 | 1.91×10^-37^ | 92 | 4A | 182.56 | 379.92 |  |
|  |  |  |  |  |  |  |  |  |  |  |  | 77 | 158 | 5.14×10^-32^ | 93 | 6D | 144.42 | 144.42 |  |
|  |  |  |  |  |  | GMI_ES_LB_11757 | 13.8 cM | SNP | Mrg08 (112.5 cM)^g^ | GMI_ES_LB_11757 | 7 | 100 | 239 | 9.91×10^-54^ | 99 | 2D | 136.81 | 136.81 |  |
|  |  |  |  |  |  |  |  |  |  |  |  | 100 | 219 | 2.66x10^-48^ | 95 | 3D | 347.05 | 347.05 |  |
|  |  |  |  |  |  |  |  |  |  |  |  | 100 | 488 | 1.68x10^-44^ | 86 | 5A | 10.49 | 431.09 |  |
|  |  |  |  |  |  | GMI_ES15_c3200 | 13.8 cM | SNP | Mrg08 (109.2 cM)^g^ | GMI_ES15_c3200_563 | 9 | 100 | 234 | 1.21×10^-52^ | 98 | 2D | 143.14 | 143.14 |  |
|  |  |  |  |  |  |  |  |  |  |  |  | 100 | 214 | 3.24×10^-47^ | 95 | 2A | 346.62 | 346.62 |  |
|  |  |  |  |  |  |  |  |  |  |  |  | 93 | 196 | 2.49×10^-42^ | 94 | 2C | 496.14 | 496.14 |  |
|  |  |  |  |  |  | GMI_ES02_c14533 | co-segregating | SNP | Mrg08 (80.2)^g^ | GMI_ES02_c14533_567 | 6 | 100 | 234 | 1.21x10^-52^ | 98 | 2D | 180.49 | 180.49 |  |
|  |  |  |  |  |  |  |  |  |  |  |  | 100 | 224 | 6.25x10^-50^ | 96 | 2C | 542.19 | 542.19 |  |
|  |  |  |  |  |  |  |  |  |  |  |  | 100 | 316 | 3.24x10^-7^ | 89 | 2A | 372.77 | 388.27 |  |
|  |  |  |  |  |  | GMI_GBS_821 | 13.8 cM | SNP | Mrg08 (112.5 cM)^g^ | GMI_GBS_821 | 22 | 100 | 4395 | 3.32x10^-23^ | 93 | 2D | - | - |  |
|  |  |  |  |  |  |  |  |  |  |  |  | 75 | 5832 | 8.35x10^-12^ | 92 | 4D | - | - |  |
|  |  |  |  |  |  |  |  |  |  |  |  | 77 | 7034 | 8.35x10-12 | 92 | 4A | - | - |  |
| *Pc45* |  | Morton, Kame, OT3060 | Gnanesh et al. (2015) | OT3019Morton, WeaverKame, OT9001OT3060 | F5:6 RILs | I05-0874-KOM16c1 (PcKMSNP1) | co-seg, 2.2 cM, co-seg | RAPD | chr 12D (66.5 cM)^f^, Mrg08 (80.2 - 81.4 cM)^g^ | I05-0874-KOM16c1 (PcKMSNP1) | 22 | 100 | 156581 | 0 | 73 | 2D | - | - |  |
|  |  |  |  |  |  |  |  |  |  |  |  | 48 | 132943 | 1.33x10^-160^ | 73 | 4C | - | - |  |
|  |  |  |  |  |  |  |  |  |  |  |  | 50 | 148502 | 1.33x10^-160^ | 73 | 5D | - | - |  |
|  |  | Cav5050 | Kebede et al. (2019) | MorganPc45, KasztanPc45 | F2:3 RILs |  |  |  |  |  |  |  |  |  |  |  |  |  |  |
|  |  |  |  |  |  |  |  |  |  |  |  |  |  |  |  |  |  |  |  |
|  |  |  |  |  |  | Avgbs2_172881.1 | co-segregating | SNP | - | Avgbs2_172881.1 | 2 | 91 | 118 | 1.45x10^-21^ | 100 | 2D | 180.00 | 180.00 |  |
|  |  |  |  |  |  |  |  |  |  |  |  | 91 | 91 | 5.79x10^-14^ | 91 | 2A | 387.64 | 387.64 |  |
| *Pc48* | | Pendek48 | Wight et al. (2004) | Pendek3948 | F2:3 RILs | umn401 | 6 cM | RFLP | KO _22_44+18 (72 cM)^d^ | CK780289 | 6 | 99 | 1270 | 2.18×10^-153^ | 98 | 4A | 284.62 | 405.95 |  |
|  |  |  |  |  |  |  |  |  |  |  |  | 99 | 1412 | 9.25×10^-152^ | 96 | 4D | 309.48 | 448.96 |  |
|  |  |  |  |  |  |  |  |  |  |  |  | 99 | 1007 | 9.26×10^-133^ | 96 | 7C | 54.27 | 54.27 |  |
|  |  |  |  |  |  | cdo1471 | 6 cM | RFLP | KO _22_44+18 (70 cM)^d^ | CN180772 | 4 | 99 | 948 | 8.81×10^-120^ | 97 | 4D | 301.50 | 301.50 |  |
|  |  |  |  |  |  |  |  |  |  |  |  | 99 | 890 | 1.22x10^-111^ | 95 | 4A | 275.92 | 275.92 |  |
|  |  |  |  |  |  |  |  |  |  |  |  | 84 | 932 | 1.01x10^-93^ | 85 | 7C | 65.79 | 65.84 |  |
|  |  |  |  |  |  | bcd1643c | 6 cM | RFLP | KO _22_44+18 (77.5 cM)^d^ | BE438817 | 3 | 82 | 561 | 9.63x10^-106^ | 92 | 4D | 306.63 | 306.63 |  |
|  |  |  |  |  |  |  |  |  |  |  |  | 82 | 541 | 2.12x10^-101^ | 91 | 4A | 281.71 | 281.71 |  |
|  |  |  |  |  |  |  |  |  |  |  |  | 82 | 816 | 1.34x10^-97^ | 87 | 7C | 58.24 | 479.67 |  |
|  |  |  |  |  |  | aco165rv | 6 cM | RFLP | KO _22_44+18 (59 cM)^d^ | CN180765 | 5 | 99 | 1162 | 0 | 96 | 7C | 52.98 | 52.98 |  |
|  |  |  |  |  |  |  |  |  |  |  |  | 99 | 1055 | 0 | 93 | 4D | 311.46 | 311.46 |  |
|  |  |  |  |  |  |  |  |  |  |  |  | 99 | 1050 | 0 | 93 | 4A | 285.75 | 285.75 |  |
|  |  | - | Klos et al. 2017 | CORE | GWAS panel | GMI_DS_LB_6135 | - | SNP | Mrg20 (122.8 cM)^g^ | GMI_DS_LB_6135 | 5 | 100 | 399 | 7.00x10-97 | 99 | 4D | 311.46 | 311.46 |  |
|  |  |  |  |  |  |  |  |  |  |  |  | 100 | 491 | 7.00x10-97 | 94 | 4A | 285.75 | 339.53 |  |
|  |  |  |  |  |  |  |  |  |  |  |  | 99 | 336 | 4.72x10-80 | 92 | 7C | 52.98 | 339.53 |  |
|  |  | - | McNish et al. 2020 | UMOF |  | avgbs_51923.1.27 | - | SNP | Mrg20 (63 cM)^h^ | avgbs_51923.1 | 1 | 100 | 125 | 3.32×10-^23^ | 98 | 4A | 269.46 | 269.46 |  |
| *Pc54* | | Pc54 | Admassu-Yimer et al. (2022) | Pc54Otana, Pc54Pc96 | F5:6 RILs | GMI_ES22_c2813_554 | - | SNP | Mrg02 (87.3cM)^g^ | GMI_ES22_c2813 | 2 | 100 | 752 | 3.95×10^-46^ | 91 | 7D | 465.11 | 494.02 |  |
|  |  |  |  |  |  |  |  |  |  |  |  | 100 | 312 | 1.06×10^-40^ | 85 | 6C | 5.79 | 5.88 |  |
|  |  |  |  |  |  | GMI_ES03_c95_413 | - | SNP | Mrg02 (84.6cM)^g^ | GMI_ES03_c95 | 4 | 100 | 479 | 9.92×10^-35^ | 98 | 7D | 462.94 | 490.42 |  |
|  |  |  |  |  |  |  |  |  |  |  |  | 100 | 364 | 9.92×10^-35^ | 94 | 6C | 7.43 | 206.50 |  |
|  |  |  |  |  |  |  |  |  |  |  |  | 100 | 398 | 3.04×10^-22^ | 82 | 6A | 20.43 | 20.69 |  |
|  |  |  |  |  |  | GMI_ES15_c15279_258 | - | SNP | Mrg02 (87.3cM)^g^ | GMI_ES15_c15279 | 2 | 100 | 1182 | 6.55×10^-91^ | 89 | 7D | 461.45 | 489.35 |  |
|  |  |  |  |  |  |  |  |  |  |  |  | 100 | 475 | 1.65×10-60 | 82 | 6C | 8.52 | 8.52 |  |
| *Pc58* | *Pc58a* | TAM O-301 | Hoffman et al. (2006), Jackson et al. (2008) | OgleTAM | F6:7 RILs, F6:9 RILs | cdo545b | 8.4 cm, 6.9 cM | RFLP | OT-32 (7.2 cM)^c^ | BE439189 | 3 | 99 | 449 | 9.60x10^-58^ | 97 | 7D | 520.06 | 520.06 |  |
|  |  |  |  |  |  |  |  |  |  |  |  | 99 | 444 | 4.08x10^-56^ | 97 | 7A | 54.15 | 54.15 |  |
|  |  |  |  |  |  |  |  |  |  |  |  | 99 | 312 | 2.12x10^-34^ | 87 | 5C | 602.51 | 602.51 |  |
|  |  |  | Hoffman et al. (2006), Jackson et al. (2008) |  | F6:7 RILs, F6:9 RILs | PSR160B | 4.5 cM, 3.9 cM | RFLP | OT-32 (4.2 cM)^c^ | PSR160 | 3 | 65 | 437 | 7.15x10^-107^ | 94 | 7A | 50.03 | 50.03 |  |
|  |  |  |  |  |  |  |  |  |  |  |  | 64 | 428 | 1.06x10^-104^ | 94 | 7D | 523.28 | 523.28 |  |
|  |  |  |  |  |  |  |  |  |  |  |  | 64 | 413 | 2.34x10^-100^ | 93 | 5C | 611.05 | 611.05 |  |
|  |  | Differential lines Pc14, 38, 53, 57, 58, 59, 61, 63 (Carson 2011) | Klos et al. (2017) | CORE | GWAS panel | GMI_GBS_94371 | 10 cM | SNP | Mrg02 (28 cM)^g^ | GMI_GBS_94371 | 2 | 100 | 125 | 3.32x10^-23^ | 98 | 7D | 511.40 | 511.40 |  |
|  |  |  |  |  |  |  |  |  |  |  |  | 95 | 68 | 5.27x10^-8^ | 76 | 7A | 64.45 | 64.45 |  |
|  | *Pc58c, Pc58a* | TAM O-301 | Hoffman et al. (2006),  Jackson et al. (2008) | OgleTAM | F6:7 RILs, F6:9 RILs | PSR637 | 3.1 cM, 0.3 cM | RFLP | OT-32 (0.0 cM)^c^ | PSR637 | 5 | 27 | 104 | 1.67x10^-16^ | 71 | 2D | 280.20 | 280.20 |  |
|  |  |  |  |  |  |  |  |  |  |  |  | 33 | 91 | 1.05x10^-12^ | 69 | 2C | 150.56 | 150.56 |  |
|  |  |  |  |  |  |  |  |  |  |  |  | 32 | 88 | 3.67x10^-12^ | 69 | 4A | 408.34 | 408.34 |  |
|  | *Pc58b* |  | Hoffman et al. (2006) |  | F6:7 RILs | RZ516D | 4.6 cM | RFLP | OT-33^c^ | AA231792 | 3 | 25 | 74 | 1.27x10^-8^ | 75 | 7D | 509.73 | 509.73 |  |
|  |  |  |  |  |  |  |  |  |  |  |  | 25 | 74 | 1.27x10^-8^ | 75 | 7A | 67.07 | 67.07 |  |
|  |  |  |  |  |  |  |  |  |  |  |  | 29 | 65 | 6.57x10^-6^ | 72 | 5C | 595.94 | 595.94 |  |
| *Pc68* | | AC Assiniboia | Chen et al. (2006) | MS68S42, AsbMN | F2:3, F7:9 RILs | CDO309 | 6.7 cM, 4.2 cM | RFLP | LG 4^c^, KO 4_12^d^ | CDO309_WHE05D11.s1 | 9 | 100 | 853 | 0 | 97 | 1D | 376.84 | 376.84 |  |
|  |  |  |  |  |  |  |  |  |  |  |  | 99 | 1282 | 0 | 83 | 7A | 4.10 | 158.32 |  |
|  |  |  |  |  |  |  |  |  |  |  |  | 72 | 520 | 1.35x10^-129^ | 92 | 3D | 469.92 | 469.92 |  |
|  |  | UFRGS8 | Klos et al. (2017) | CORE | GWAS panel | Avgbs_228849 | - | SNP | Mrg19 (70.1 cM)^g^ | avgbs_228849 | 4 | 86 | 107 | 2.55x10^-18^ | 98 | 3D | 470.13 | 470.13 |  |
|  |  |  |  |  |  |  |  |  |  |  |  | 86 | 72 | 4.33x10^-9^ | 85 | 7C | 17.65 | 17.65 |  |
|  |  |  |  |  |  |  |  |  |  |  |  | 83 | 68 | 5.27x10^-8^ | 84 | 2C | 554.36 | 554.36 |  |
|  |  | - |  |  |  | UMN214A | - | RFLP | Mrg19^g^ | CK780254 | 7 | 100 | 5165 | 0 | 82 | 3D | 450.84 | 479.79 |  |
|  |  |  |  |  |  |  |  |  |  |  |  | 100 | 3217 | 0 | 78 | 1D | 372.08 | 394.10 |  |
|  |  |  |  |  |  |  |  |  |  |  |  | 100 | 4107 | 0 | 84 | Un | 15.64 | 15.67 |  |
| *Pc91* | | HiFi | McCartney et al. (2011) | CDC Sol-FiHiFi | F7:8 RILs | oPt-0350 | 1.2 cM | DArT | KOD_1_3_38_X3^e^, KOD_1_3_38_break-point^e^, KOD_1_3_38_X1^e^ | oPt-0350-cdc | 2 | 100 | 450 | 1.50x10^-46^ | 80 | 1A | 419.73 | 419.81 |  |
|  |  |  |  |  |  |  |  |  |  |  |  |  |  |  |  |  |  |  |  |
|  |  | Stainless | Gnanesh et al. (2013) | MorganStainless | F2 |  | 1.9 cM |  | 7C-17A (71 cM)^f^ |  |  |  |  |  |  |  |  |  |  |
|  |  | Stainless |  | BetaniaStainless | F2 |  | co-seg |  |  |  |  | 100 | 397 | 2.90x10^-30^ | 80 | 2D | 52.42 | 52.48 |  |
|  |  | CDCMorrison |  | MorganCDCMorrison | F2 |  |  |  |  |  |  |  |  |  |  |  |  |  |  |
|  |  | HiFi |  | CDC Sol-Fi HiFi | F7 RIL |  |  |  |  |  |  |  |  |  |  |  |  |  |  |
|  |  | Amagalon | Rooney et al. (1994) | AmaSt1, AmaOgle1 | BC1F6 | UMN145 | 4.5 cM | RFLP | Chromsome 18^a^ | UMN145 | 3 | 93 | 836 | 0 | 100 | 6C | 605.53 | 605.53 |  |
|  |  |  |  |  |  |  |  |  |  |  |  | 92 | 590 | 1.32x10-148 | 87 | 6A | 285.03 | 285.03 |  |
|  |  |  |  |  |  |  |  |  |  |  |  | 92 | 582 | 1.96x10-146 | 86 | 6D | 246.92 | 246.92 |  |
|  |  | Differential lines Pc91 (Carson 2011) | Klos et al. (2017) | CORE | GWAS panel | GMI_ES03_c2277_336 | - | SNP | Mrg18 (67.7cM)^g^ | GMI_ES03_c2277_336 | 1 | 100 | 239 | 9.91x10^-54^ | 99 | 1A | 387.53 | 387.53 |  |
| *Pc92* | | Obee/Midsouth | Rooney et al. (1994) | ObMidSt1, ObMidOgle1 | F2 RILs | OG176 | 13.6 cM | RFLP | - | - | - | - | - | - | - | - | - | - |  |
| *Pc94* | | S42 | Chong et al. (2004) | CalS42, MS68S42 | F2:3 RILs | AF94a | 0.9 cM, 3.4 cM | AFLP | - | AY084050.1 | 22 | 95 | 4836 | 0 | 73 | 1D | 3.04 | 473.30 |  |
|  |  |  |  |  |  |  |  |  |  |  |  | 90 | 4238 | 0 | 69 | 7D | 60.50 | 523.77 |  |
|  |  |  |  |  |  |  |  |  |  |  |  | 90 | 1232 | 0 | 68 | 5C | 38.37 | 562.61 |  |
|  |  |  | Chen et al. (2007) | AsbS42, CalS42 |  | Pc94-SNP1a | 2.1 cM, 5.4 cM | SNP |  | Pc94-SNP1a | 1 | 100 | 81 | 1.46x10^-11^ | 93 | 7D | 485.00 | 485.00 |  |
| *Pc98* | | CAV 1979 | Zhao et al. (2020b) | Pc98Bingo | F2:3 RILs | GMI_ES22_c3052_382_kom399 | 0.9 cM | SNP | Mrg20 (32 cM)^g^ | GMI_ES22_c3052_382 | 5 | 100 | 239 | 9.91x10^-54^ | 99 | Un | 34.01 | 34.01 |  |
|  |  |  |  |  |  |  |  |  |  |  |  | 100 | 239 | 9.91x10^-54^ | 99 | 4A | 463.19 | 463.19 |  |
|  |  |  |  |  |  |  |  |  |  |  |  | 90 | 131 | 2.05x10^-24^ | 79 | 6C | 3.33 | 3.33 |  |
|  |  |  |  |  |  | GMI_ES14_lrc18344_662_kom398 | 0.3 cM | SNP | Mrg20 (29 cM)^g^ | GMI_ES14_lrc18344_662 | 2 | 100 | 223 | 2.18x10^-49^ | 97 | 4D | 407.11 | 407.12 |  |
|  |  |  |  |  |  |  |  |  |  |  |  | 100 | 207 | 4.81x10^-45^ | 94 | 4A | 456.84 | 456.84 |  |
|  |  |  |  | Pc98Kasztan | F2:3 RILs | GMI_DS_LB_6017_kom367 | 0.7 cM | SNP | Mrg20 (22cM)^g^ | GMI_DS_LB_6017 | 4 | 100 | 622 | 7.00×10^-97^ | 82 | 4A | 451.32 | 451.33 |  |
|  |  |  |  |  |  |  |  |  |  |  |  | 100 | 1365 | 2.29×10^-90^ | 85 | 4D | 402.22 | 447.29 |  |
|  |  |  |  |  |  |  |  |  |  |  |  | 97 | 157 | 3.40×10^-31^ | 78 | 3D | 135.82 | 135.82 |  |
|  |  |  |  |  |  | avgbs2_153634.1.59_kom410 | 0.3 cM | SNP | Mrg20 (33 cM)^g^ | avgbs2_153634.1 | 3 | 100 | 221 | 6.01x10^-20^ | 91 | 4D | 97.15 | 410.36 |  |
|  |  |  |  |  |  |  |  |  |  |  |  | 100 | 113 | 6.01x10^-20^ | 92 | 4A | 461.23 | 461.23 |  |
|  |  |  |  |  |  |  |  |  |  |  |  | 100 | 98 | 3.79x10^-16^ | 87 | 4C | 219.22 | 219.22 |  |
| The BLAST threshold parameter was established at 10−5, with only hits with E values below this cutoff considered as significant. Top three hits presented | | | | | | | | | | | | | | | | | | |  |
|  |  |  |  |  |  |  |  |  |  |  |  |  |  |  |  |  |  |  |  |
| Pedigree of population ID^1^: | | | | | | | | | | | | | | | | | | |  |
| AmaOgle1 ((Amagalon (PI 497742 (CI 8330/CW-57) x (Ogle-1 (Brave//Tyler/Egdolon 23))) | | | | | | | | | | | | | | | | | | |  |
| AmaSt1 ((Amagalon (CI 8330/CW-57) x (Starter-1 (DaF/3/Garland/Bur-nettV/Diana/CI 8344/4/Noble))) | | | | | | | | | | | | | | | | | | |  |
| AsbMN ((AC Assiniboia (90GC144/7*Robert) x (MN841801 (Florad/Coker 58-73/CI7558//Black Mesdag/Aberdeen 101))) | | | | | | | | | | | | | | | | | | |  |
| AsbS42 ((AC Assiniboia(90GC144/7*Robert) x (S42(Sun II*9/RL1697))) | | | | | | | | | | | | | | | | | | |  |
| BetaniaStainless ((SW Betania(SW 151-7-95/SW 52-95) x (Stainless(ND931475/AC Assiniboia//HiFi))) | | | | | | | | | | | | | | | | | | |  |
| CalS42 ((Calibre (Gemini/Clintford) x (S42 (Sun II*9/RL1697))) | | | | | | | | | | | | | | | | | | |  |
| CDC Sol-FiHiFi ((CDC Sol-Fi (N979-5-1/OT366) x (HiFi (Amagalon/4/M23/RL3038//Otana/3/Froker/RL3038//RL3038/Hudson/5/MN78142/4/W80-20/3/Hudson/Lang//Dal))) | | | | | | | | | | | | | | | | | | |  |
| CelerSTH9210 ((Celer (Góral/KR-KOR)) x (STH9210 ((STH212/STH13817)/(STH214/STH13827))) | | | | | | | | | | | | | | | | | | |  |
| CORE (Collaborative Oat Research Enterprise population) - 631 oat lines(Tinker et al., 2016) | | | | | | | | | | | | | | | | | | |  |
| D526BC ((D526 (Lang*3/CIav9286) x (Lang (Tyler/Orbit))) | | | | | | | | | | | | | | | | | | |  |
| KasztanPc45 ((Kasztan (Dawid/CHD 1685/84) x (Pc45 (Pendek*4/CAV5050))) | | | | | | | | | | | | | | | | | | |  |
| MedMN ((AC Medallion (90GC144/7*Dumont) x (MN841801 (Florad/Coker 58-73/CI7558//Black Mesdag/Aberdeen 101))) | | | | | | | | | | | | | | | | | | |  |
| MorganCDCMorrison ((AC Morgan(OT526/OT763) x (CDC Morrison(CDC Sol-Fi/HiFi))) | | | | | | | | | | | | | | | | | | |  |
| MorganPc45 ((AC Morgan(OT526//Fidler/Cascade) x (Pc45 (Pendek*4/CAV5050))) | | | | | | | | | | | | | | | | | | |  |
| MorganStainless ((AC Morgan(OT526/OT763) x (Stainless(ND931475/AC Assiniboia//HiFi))) | | | | | | | | | | | | | | | | | | |  |
| MS68S42 ((Makuru-Sun II-Pc68 (Makuru*2//CAV4904/2*Sun II) x (S42 (Sun II*9/RL1697))) | | | | | | | | | | | | | | | | | | |  |
| ObMidOgle1 (PI 497874 (Obee/Midsouth) x (Ogle-1 (Brave//Tyler/Egdolon 23)) | | | | | | | | | | | | | | | | | | |  |
| ObMidSt1 ((PI 497874 (Obee/Midsouth) x (Starter-1 (DaF/3/Garland/Bur-nettV/Diana/CI 8344/4/Noble))) | | | | | | | | | | | | | | | | | | |  |
| OgleTAM ((Ogle (Brave//Tyler/Egdolon 23) x (TAM O-301(AB555/3/ORA/63C3868-4-2//ORA/3*PI295919))) | | | | | | | | | | | | | | | | | | |  |
| OT3019Morton ((OT3019 (W97601/SA96482) x (Morton (ND880922/B605X))) | | | | | | | | | | | | | | | | | | |  |
| OT328Du ((OT328 (S79 107/Cascade) x (Dumont (Harmon HAM/Double Cross7))) | | | | | | | | | | | | | | | | | | |  |
| OT9001OT3060 ((OT9001 (SW96290/SW00263) x (OT3060 (OT3019/Morton))) | | | | | | | | | | | | | | | | | | |  |
| Pc53Otana ((Pc53 (Clinton/6-112-1-15) x (Otana (CI 5345/Zanster (CI 5345/2*Overland))) | | | | | | | | | | | | | | | | | | |  |
| Pc53Pc50 ((Pc53 (Clinton/6-112-1-15) x (Pc50 (CAV 2643/4*Pendek))) | | | | | | | | | | | | | | | | | | |  |
| Pc54Otana ((Pc54(Pendek*2/CAV 1832) x (Otana(CI 5345/Zanster (CI 5345/2*Overland))) | | | | | | | | | | | | | | | | | | |  |
| Pc54Pc96 ((Pc54(Pendek*2/CAV 1832) x (Pc96 (RL1730(Makuru/MG 85039)))) | | | | | | | | | | | | | | | | | | |  |
| Pc98Bingo ((Pc98 (Harmon*2/CAV 1979) x (Bingo (STH 214 (Gramena/3/Dukat//Flamingsnowa/Swan)/STH 13827 (Bandicot/3/Kondradin//Flamingsnowa/Swan))) | | | | | | | | | | | | | | | | | | |  |
| Pc98Kasztan ((Pc98(Harmon*2/CAV 1979) x (Kasztan(Dawid/CHD 1685/84))) | | | | | | | | | | | | | | | | | | |  |
| Pendek3948 ((Pendek39 (CAV 5165 (F366)/4*Pendek)) x (Pendek48 ((Pendek*2/A. sterilis CAV 5401 (F158))) | | | | | | | | | | | | | | | | | | |  |
| Pendek4838 ((Pendek48 (Pendek*2/ A. sterilis CAV 5401 (F158)) x (Pendek38 (CAV 2648-4 (CW491-4)/4*Pendek))) | | | | | | | | | | | | | | | | | | |  |
| UMOF (University of Minnesota Oat Founder population) - 256 oat lines (McNish et al. 2022) | | | | | | | | | | | | | | | | | | |  |
| WeaverKame ((CDC Weaver (OT369/W95116) x (Kame (B605X/(Dane/Newdak))) | | | | | | | | | | | | | | | | | | |  |
|  |  |  |  |  |  |  |  |  |  |  |  |  |  |  |  |  |  |  |  |
| Reference for comparative map ID^2^: | | | | | | | | | | | | | | | | | | |  |
| ^a^Kanota monosomic - Jellen et al. (1993) | | | | | | | | | | | | | | | | | | |  |
| ^b^KxO 1995 - O’Donoughue et al. (1995) | | | | | | | | | | | | | | | | | | |  |
| ^c^OglexTAM 2001 - Portyanko et al. (2001) | | | | | | | | | | | | | | | | | | |  |
| ^d^KxO 2003 - Wight et al. (2003) | | | | | | | | | | | | | | | | | | |  |
| ^e^KxO 2009 - Tinker et al. (2009) | | | | | | | | | | | | | | | | | | |  |
| ^f^Consensus 2013 - Oliver et al. (2013) | | | | | | | | | | | | | | | | | | |  |
| ^g^Consensus 2016 - Chaffinet al. (2016) | | | | | | | | | | | | | | | | | | |  |
| ^h^Consensus 2018 - Bekele et al. (2018) | | | | | | | | | | | | | | | | | | |  |
